# Reefal regions were biodiversity hotspots throughout the Phanerozoic

**DOI:** 10.1126/sciadv.adv9793

**Published:** 2025-11-05

**Authors:** Roger A. Close, Roger B. J. Benson, Wolfgang Kiessling, Erin E. Saupe

**Affiliations:** ^1^ Department of Earth Sciences, University of Oxford, Oxford OX1 3AN, UK.; ^2^ Division of Paleontology, American Museum of Natural History, New York, NY 10024-5102, USA.; ^3^ Department of Geography and Geosciences, Friedrich-Alexander-Universität, Erlangen 91054, Germany.

## Abstract

Reefs are important hotspots of marine biodiversity today and have acted as cradles of diversification in the geological past. However, we know little about how the diversity of reef-supporting regions varied through deep time, and how this differed from other regions. We quantified regional diversity patterns in reef-supporting and non–reef-supporting regions in the fossil record of Phanerozoic marine invertebrates. Diversity in reef-supporting regions is on average two- to threefold higher than in non–reef-supporting regions and has been remarkably stable over timescales of tens to hundreds of millions of years. This signal is present in both reefal and non-reefal facies within reef-supporting regions, suggesting that reefs enriched diversity in surrounding environments. Sepkoski’s “Modern Fauna,” an assemblage of higher taxa that includes gastropods, bivalves, and echinoids, has been a key component of reef-supporting regions since the Paleozoic, contrasting with its later rise to dominance in non–reef-supporting regions during the later Mesozoic-Cenozoic.

## Teaser

Geographic regions that support reefal environments have been key hotspots of marine animal diversity for over 400 million years.

## INTRODUCTION

Biodiversity varies considerably among environments on Earth today. In the oceans, tropical coastal zones are approximately twice as rich as temperate coastal zones (*1*, *2*), and coral-algal reefs host a disproportionate share of extant biodiversity, supporting an estimated one-third of all marine species despite occupying only 0.1% of Earth’s surface (*1*, *3*). Diversity also varies among environments in the fossil record [e.g., (*4*, *5*)]. Regionally, tropical shallow-water habitats, especially reefs, have been sites of net origination, exporting species to other environments and to higher latitudes in deep time (*6*, *7*). The occurrence of high diversity within reefal environments is a persistent, first-order observation of paleobiology [e.g., (*8*–*14*)]. Geographic regions that support reefal environments may therefore be characterized by different diversity dynamics to those that do not support reefs on Phanerozoic timescales. These differences may be central to explaining large-scale patterns in the taxonomic composition and richness of the marine biota [e.g., (*15*, *16*)], but they remain poorly understood, and no study has directly contrasted diversity within reefal and non-reefal environments over the Phanerozoic.

Patterns of marine animal diversity through the Phanerozoic have long been analyzed at a notionally “global” scale. However, the fossil record for any time in Earth’s history is never truly global in scope, with sampled geographic regions varying substantially in number, size, location, and environmental context. Therefore, some of the apparent variation in global diversity through time may be structured by variation in the geographic and environmental scope of the sampled fossil record (*5*, *8*, *10*, *17*–*21*). Spatially and environmentally explicit analyses can overcome these biases and have great potential for uncovering variation in the geographic and environmental structure of diversity through time. Here, we quantify changes in diversity for reef-supporting versus non–reef-supporting regions over the Phanerozoic, to identify hotspots of biodiversity on multimillion-year timescales and assess spatial variation in macroevolutionary patterns.

## RESULTS

### Fossil occurrence data and reconstruction of diversity among environments

We reconstructed patterns of local- to regional-scale diversity at genus level for Phanerozoic marine animals using occurrence data from the Paleobiology Database (*22*) (see Materials and Methods). We present results for a range of sifting criteria but focus on those that exclude deposits that are unlithified or poorly lithified and sieved, or which lack information about lithification style (see Materials and Methods). To estimate diversity at smaller spatiotemporal scales, we tallied counts of genera per collection (i.e., spatially and temporally resolved fossil localities; a measure of α diversity or within-community/local richness) and per geological formation (see Materials and Methods).

**Fig. 1.**
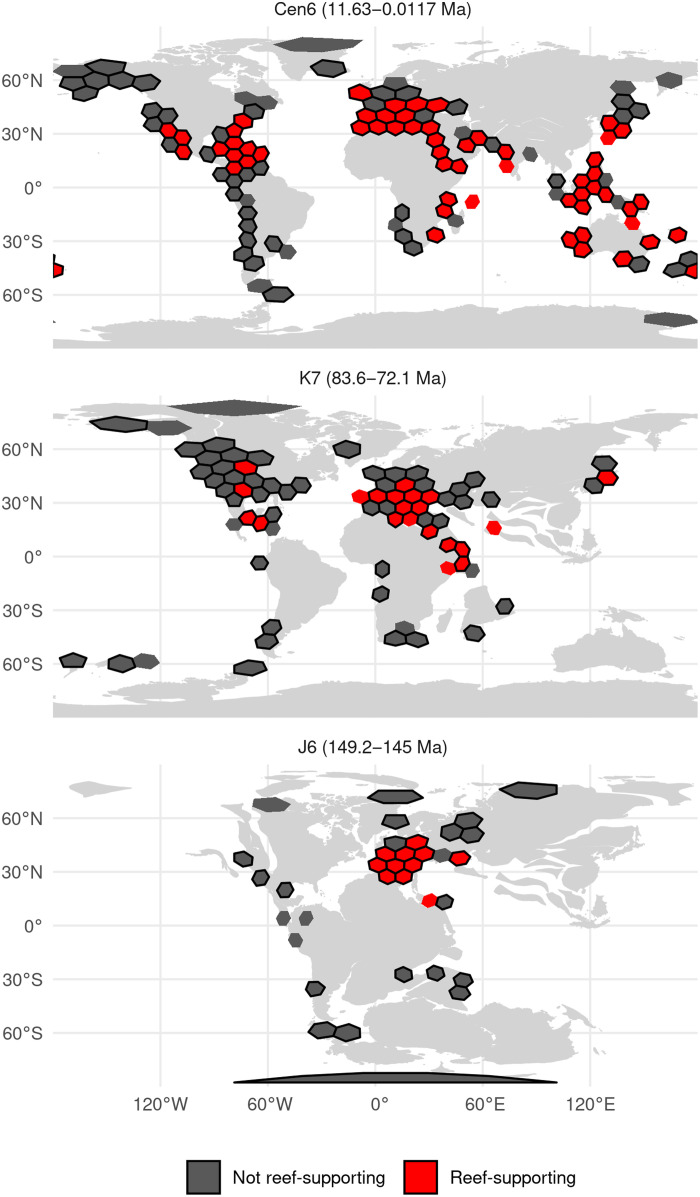
Paleogeographic distributions of reef-supporting (red) and non–reef-supporting (gray) regions (equal-area hexagonal/pentagonal grid cells with 1000-km spacings) for three of the 49 equal-length time intervals analyzed (Cen6, K7, and J6). Black borders denote grid cells that meet our quality criteria (i.e., cells containing at least 10 collections and two references), while those without black borders are those that do not. Cen6 [11.62 to 0.0117 million years (Ma)] = Messinian, Tortonian, Zanclean, Piacenzian, Gelasian, Calabrian, Middle Pleistocene, and Late Pleistocene; K7 (83.6 to 72.1 Ma) = Campanian; J6 (152.1 to 145Ma) = Tithonian. Paleomaps from the PALEOMAP project, with plate reconstructions via rgplates R package (CC BY 4.0 license; https://creativecommons.org/licenses/by/4.0/) (*45*). See fig. S1 for maps of all equal-length bins.

To obtain regional-scale diversity estimates while standardizing for spatial sampling, we binned fossil localities into equal-area hexagonal/pentagonal grid cells with 100-, 500-, and 1000-km spacings (our main-text results focus on 1000-km spacings; Fig. 1 and fig. S1; see Materials and Methods), before computing diversity for each grid cell using shareholder quorum subsampling [SQS (*16*, *23*); see Materials and Methods].

To analyze counts of genera per fossil collection, we analyzed diversity patterns for reefal and non-reefal environments (facies). However, we classified grid cells and formations as either “reef-supporting” or “non–reef-supporting” based on the presence or absence of reefal collections (Fig. 1 and figs. S1 and S2; this approach agrees with information in the PaleoReefs Database (PARED): fig. S3; see Materials and Methods). Data for reefs are substantially less abundant than for most other facies (fig. S4), consistent with the observation that modern-day reefs account for only 0.1% of the Earth’s surface by area (*1*), which imposes limits on our ability to infer regional-scale diversity patterns for reefal and non-reefal facies separately through time. Classifying grid cells based on the presence or absence of reefs is valid if diversity within reef-supporting regions is high compared to that of other regions that do not support reefs, independent (or partially independent) of the other facies represented by individual collections within those regions. We tested this hypothesis by quantifying diversity patterns separately for (i) reefal collections within reef-supporting grid cells (i.e., cells that have yielded collections representing reefal facies); (ii) non-reefal collections within reef-supporting grid cells; and (iii) non-reefal collections in non–reef-supporting cells (i.e., grid cells that have not yielded reefal collections). We held sampling intensity within grid cells constant by subsampling to equal counts of collections, using quotas of 20 and 40. We found that non-reefal collections from reef-supporting cells have similar mean diversities to those of reefal collections and higher diversities than those of cells that lack reefs, supporting the designation of a cell as “reef-supporting” if containing any reefal facies (fig. S5 and table S1). At all quotas and cell sizes, both reefal and non-reefal facies in reef-supporting cells are significantly more diverse than non-reefal facies in non–reef-supporting cells. By contrast, the diversity of reefal facies in reef-supporting cells is not significantly different from non-reefal facies in reef-supporting cells for all quotas and cell sizes, except quota = 20 using cell size 500 km. Various hypotheses may explain why non-reefal environments hosted higher diversity in reef-supporting regions than elsewhere, including environmental heterogeneity (*7*), conducive climate [for example, tropical coastal zones are more diverse than temperate zones today, even outside of reefs (*1*)], or because reefs are net exporters of species to surrounding environments (*7*).

### Patterns of diversity among reef-supporting and non–reef-supporting regions

**Fig. 2.**
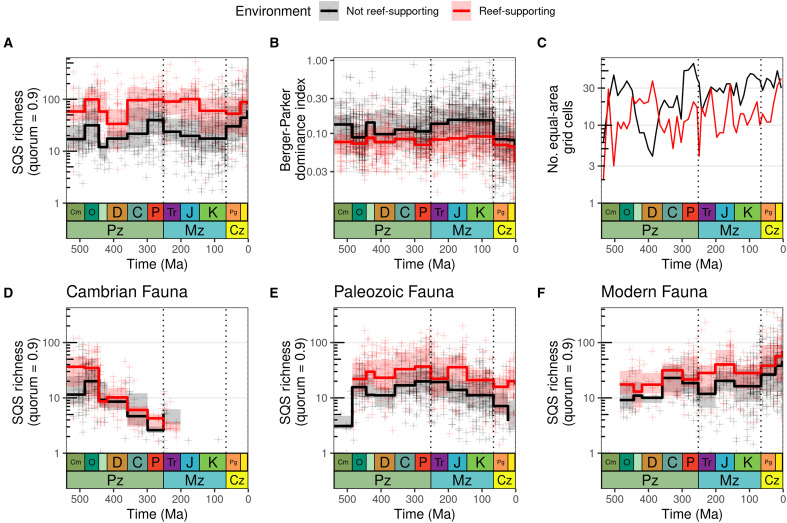
Differences in Phanerozoic marine invertebrate animal diversity patterns between reef-supporting (red) and non–reef-supporting (black) regions (equal-area hexagonal/pentagonal grid cells with spacings of 1000 km for all panels in this figure). Excluding collections explicitly identified as representing unlithified or poorly lithified-and-sieved deposits and also excluding collections that have no information about lithification style (see fig. S10 for patterns using other sifting criteria). Dotted lines represent boundaries between geological eras. Note logarithmic *y* axes. For (A), (B), (D), and (F), crosses represent SQS diversity estimates for individual grid cell regions within equal-length time bins, while summary trend lines represent medians and interquartile ranges of regional diversity for geological periods (i.e., summarizing patterns for equal-length data points across longer timescales). (**A**) Spatially standardized Phanerozoic marine animal diversity, contrasting patterns for reef-supporting and non–reef-supporting regions. Note that in reef-supporting regions, levels of diversity have been broadly similar since the Ordovician, with no evidence for long-term, secular trends. In non–reef-supporting regions, by contrast, levels of diversity were similar from the Ordovician to the latest Cretaceous, when diversity rose fairly rapidly to a new, higher level that was sustained through the Cenozoic. However, this K/Pg increase is strongly associated with gastropods and unlithified sediments (see fig. S9). (**B**) Evenness, estimated using the Berger-Parker dominance index (*49*), in reef-supporting and non–reef-supporting grid cells. (**C**) Counts of reef-supporting and non–reef-supporting cells through the Phanerozoic, using equal-length time bins. (**D** to **F**) Patterns for Sepkoski’s evolutionary faunas. (D) Cambrian Fauna (Trilobita, Linguliformea, Graptolithina, and Conodonta); (E) Paleozoic Fauna (Anthozoa, Ostracoda, Rhynchonelliformea, Cephalopoda, and Crinoidea); (F) Modern Fauna (Bryozoa, Bivalvia, Gastropoda, Echinoidea, and Chondrichthyes).

We find that reef-supporting regions hosted between two- to threefold higher levels of diversity, on average, than non–reef-supporting regions throughout the Phanerozoic, depending on grid cell size, pre– or post–Cretaceous-Paleogene time interval, and sifting criteria (Fig. 2A and fig. S6). For Wilcox tests of differences and effect sizes quantified as *r* values, see tables S2 and S3; additional spatial scales are shown in fig. S7, and patterns for data binned by geological stage are shown in fig. S8; patterns of diversity through time are visually identical for alternate tectonic plate models; see Materials and Methods), all of which are qualitatively similar to our focal results. Higher diversity in reef-supporting regions is returned despite the fact that reef-supporting regions are less numerous and have a more restricted spatial distribution compared to non-reefal regions: Non–reef-supporting cells outnumbered reef-supporting cells through much of the Phanerozoic (except during the Silurian–Devonian; Fig. 2C), although counts of reef-supporting cells increase toward the present-day and marginally exceed non–reef-supporting cells in the most recent time bin.

Diversity within reef-supporting regions probably reached modern levels by the Ordovician and did not experience substantial step changes or sustained increases over the remainder of the Phanerozoic (Fig. 2A). Average levels of diversity in non–reef-supporting regions increased across the K/Pg boundary to approximately twice those attained throughout most of the preceding Phanerozoic, although a peak of comparable magnitude was reached earlier in the Ordovician (Fig. 2A and fig. S9A; see tables S3 and S4 for effect sizes). As a result, diversity within reef-supporting regions was approximately three times higher than non–reef-supporting regions on average during the Paleozoic-Mesozoic but only approximately twice as high during the Cenozoic (table S4). It is possible that this increase in non–reef-supporting regions after the K/Pg may be exaggerated by the presence of less–well-lithified deposits in the dataset: Only a modest increase is seen when analyzing lithified data only, but a pronounced increase is seen when including all data regardless of lithification style (fig. S10 and table S4). The Cenozoic radiation of gastropods may also play a role in the observed increase (see below). Wilcoxon statistical tests show a highly significant increase in diversity for non–reef-supporting regions from the Paleozoic/Mesozoic into the Cenozoic when including collections that lack metadata on lithification, but this is weaker when lithified-only data are analyzed (fig. S9A and table S4). Although controlling for lithification style is justified on the basis of the findings of previous studies (*24*, *25*), restricting the analysis to lithified data only disproportionately reduces the amount of available data for the Cenozoic, especially in siliciclastic settings (carbonate lithologies are much more likely to be lithified than siliciclastic lithologies; fig. S11). When splitting the underlying occurrence data into carbonate and siliciclastic lithologies before diversity estimation, the difference in diversity between reef-supporting and non–reef-supporting regions is more pronounced within carbonate lithologies, while reef-supporting regions within carbonate lithologies are more diverse than in siliciclastic regions (fig. S12).

**Fig. 3.**
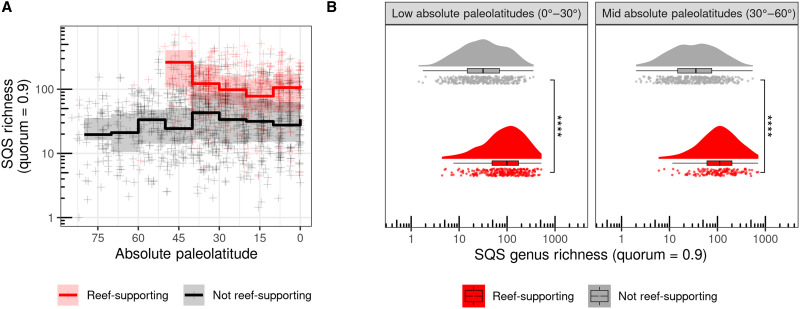
The effect of paleolatitude on diversity within reef-supporting and non–reef-supporting regions. (**A**) Latitudinal gradients of Phanerozoic marine invertebrate animal diversity, showing separate trends across paleolatitudes for reef-supporting (red) and non–reef-supporting (black) regions (equal-area hexagonal/pentagonal grid cells with spacings of 100, 500, and 1000 km). Crosses represent SQS diversity estimates for individual grid cell regions within equal-length time bins, while summary trend lines represent medians and interquartile ranges of regional diversity for 10 degree paleolatitudinal bins. PaleoDB fossil collections that had been explicitly identified as representing unlithified or poorly lithified-and-sieved deposits were excluded. Note the logarithmic *y* axis. Diversity is significantly higher in reef-supporting regions than in non–reef-supporting regions, even at low paleolatitudes, indicating that this effect is not solely driven by higher diversity in all tropical low-paleolatitude regions. (**B**) Distributions of SQS diversity (quorum = 0.9) within reef-supporting (red) and non–reef-supporting (gray) regions (equal-area hexagonal/pentagonal grid cells with 100-, 500-, and 1000-km spacings) between low (0° to 30°) and mid (30° to 60°) absolute paleolatitude zones. Diversity is consistently higher in reef-supporting regions than in non–reef-supporting regions, regardless of paleolatitude zones. Each plot shows three ways of visualizing the data: individual data points with jitter, a boxplot (line denotes the median value, the hinges of the box correspond to the interquartile range, and whiskers extend from the hinges to the smallest/largest values at most 1.5 * interquartile range of the hinge), and a density plot using a Gaussian kernel with a smoothing bandwidth of 1. Statistical significance for Wilcoxon tests between groups is indicated by ****P ≤ 0.0001.

Reef-supporting regions are significantly more diverse than non–reef-supporting regions even at low paleolatitudes, demonstrating that this effect is not due solely to higher diversity in low-latitude tropical regions (Fig. 3 and figs. S13 and S14). The finding that reef-supporting regions were more diverse than non–reef-supporting regions is also consistent regardless of grid cell size choice, but differences become more pronounced as cell sizes increase (fig. S7 and tables S2 and S3), suggesting that reef-supporting regions attain high diversity in part through elevated β diversity. Nevertheless, even counts of genera per collection and per formation exhibit similar trends to those observed in reef-supporting versus non–reef-supporting cells, meaning that reefal collections and reef-supporting formations are more diverse than non-reefal collections and non–reef-supporting formations, and both are nontrending when only lithified deposits are analyzed (figs. S15 and S16).

**Fig. 4.**
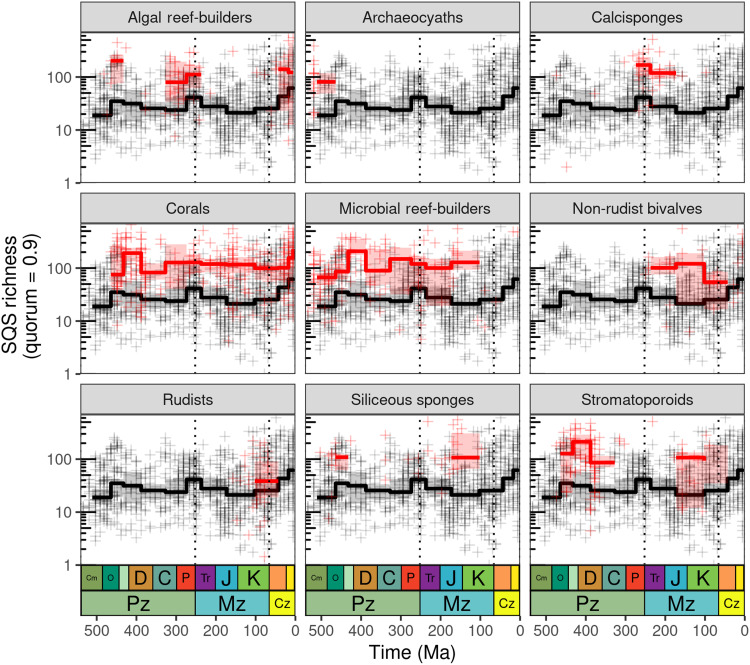
Differences in diversity patterns (SQS, quorum = 0.9) for marine invertebrate animals between reef-supporting (red) and non–reef-supporting (black) regions (equal-area hexagonal/pentagonal grid cells with 1000-km spacings), associating regions with the major kinds of reef-building organisms present within them (see fig. S19 for additional reef-building organisms). Reef-supporting cells were identified as being associated with each particular kind of reef-building organism using the palaeocoordinates of reef sites listed in the PARED PaleoReefs database (*57*) (note that reef-supporting grid cells can appear in more than one panel if they contain more than one type of reef-building organism). Diversity estimates in reef-supporting regions are for marine invertebrates as a whole, not just reef-building organisms (and the diversity of microbial or algal reef-builders themselves was not estimated). Note logarithmic *y* axes. Crosses represent SQS diversity estimates for individual grid cell regions within equal-length time bins, while summary trend lines represent medians and interquartile ranges of regional diversity for geological periods (i.e., summarizing patterns for equal-length data points across longer timescales). Dashed lines represent boundaries between geological eras. PaleoDB collection data excludes those identified as unlithified and poorly lithified-and-sieved deposits but includes collections with no information on lithification style.

Higher overall diversity in reef-supporting regions does not appear to result from markedly higher richness of individual clades (fig. S17) but rather from the summation of modest differences in diversity across a wide range of major groups, such as brachiopods, bivalves, and gastropods, with a greater number of higher taxa in each reef-supporting region (fig. S18). Although reef-supporting regions host markedly higher diversity of clades such as corals, sponges, and sea lilies, the overall diversity dynamics of other groups are superficially similar, and even non–reef-building organisms have higher diversity within reef-supporting regions (fig. S17). Parsing out overall marine invertebrate diversity patterns for reef-supporting regions according to the types of reef-building organisms that are associated with the reefs in each cell (according to the PARED database: see Materials and Methods; Fig. 4 and fig. S19) shows that reef-supporting cells containing corals are the most consistently diverse (Ordovician-present), followed by those containing algal/microbial reef-builders (Cambrian-Cretaceous; note that we only estimated metazoan diversity, even if PARED indicates that a reef-supporting region contains nonmetazoan reef-building organisms), while stromatoporoids were important in the Paleozoic and Jurassic-Cretaceous. Reef-supporting regions also have systematically more even occurrence-frequency distributions than non–reef-supporting regions as measured by the Berger-Parker dominance index (Fig. 2B), possibly reflecting the different structures of these ecological communities. Non–reef-supporting regions show a substantial increase in evenness over the latest-Cretaceous/Cenozoic interval, consistent with (*26*) (Fig. 2B).

Across all three of Sepkoski’s “evolutionary faunas” (*27*), diversity is higher in reef-supporting than in non–reef-supporting regions (Fig. 2, D to F). However, the diversity dynamics of Sepkoski’s “Modern Fauna” (*27*, *28*), which includes groups such as gastropods, bivalves, and echinoids, differ between reef-supporting and non–reef-supporting regions (Fig. 2F; see also fig. S17). The Modern Fauna became an important component of reef-supporting regions during the Paleozoic, showing only a weakly increasing mean diversity trajectory from the Carboniferous [358.9 million years (Ma)] onward (Fig. 2F and fig. S20F). The increase in diversity of the Modern Fauna over the K/Pg boundary interval is more pronounced in non-reefal regions (Fig. 2F and fig. S20F). The Paleozoic Fauna, primarily comprising articulate brachiopods, crinoids, corals, cephalopods, and bryozoans, had higher average diversity in reef-supporting regions than in non–reef-supporting regions (Fig. 2E). Diversity levels for the Paleozoic Fauna in both reef- and non–reef-supporting regions, however, remained more or less stable through the Paleozoic, followed by a gradual decrease through the Mesozoic and Cenozoic and a marked drop after the end-Cretaceous mass extinction, especially in non–reef-supporting regions. Despite these differential faunal dynamics, however, diversity for marine animals as a whole fluctuated around two different, but relatively unchanging, average levels in both reef-supporting and non–reef-supporting regions over much of the Phanerozoic (Fig. 2A).

The increase in diversity of the Modern Fauna over the K/Pg boundary interval is more pronounced in non-reefal regions (Fig. 2F). Much of this shift is driven by increases in gastropod diversity, which were proportionally greater in non-reefal than in reefal regions from the Maastrichtian onward (figs. S9B and S17 and table S5). The effect of this radiation on diversity patterns, however, is difficult to disentangle from the effects of changing lithification styles (fig. S21), because the availability of lithified deposits diminished over this interval (figs. S9A and S11). A large fraction of extant gastropod richness is concentrated in the very small “micromollusc” size range, which would be more easily recovered from unlithified sediments (*29*, *30*).

## DISCUSSION

We show that levels of richness were nondirectional in both reef-supporting and non–reef-supporting regions over an interval of 400 Ma. Although this seemingly contradicts classical hypotheses of directional long-term increasing diversity throughout geological time [e.g., (*27*, *31*)], it is conceivable that changes in global faunal provinciality or the total extent of shallow marine habitats could drive patterns different to those observed at regional spatial scales, although see (*10*, *18*). Our finding that reef-supporting regions supported high levels of diversity that did not change systematically over long intervals of geological time complements existing characterizations of boom-and-bust patterns of reef crises (*32*) over shorter time intervals, prompted by poorly understood threshold effects. Our analyses on a shorter temporal scale reveal the fluctuations in reef-supporting diversity in step with crises such as the end-Permian (*32*), although these may be less pronounced at local to regional scales than they would have been at global scales (figs. S20, S22, and S23; see also counts of reef-supporting cells in Fig. 2C). Nevertheless, when observed over tens to hundreds of millions of years, these environments fluctuated around a relatively unchanging average level of diversity (Fig. 2A).

Our study demonstrates that reef-supporting regions have hosted a disproportionate fraction of marine biodiversity throughout the Phanerozoic, despite profound changes in the taxonomic composition of reef-builders over this interval (*33*). This result is consistent with previous studies that found statistical evidence for the presence or prevalence of reefs being associated with high levels of diversity, either at notionally global (*8*) or regional scales (*10*). These findings may also be consistent with previous work showing that reefs and shallow-water tropical environments during the Phanerozoic were sites of high origination and acted as cradles of diversity that exported species to other environments (*7*). Foundational studies of paleobiology suggest that the groups comprising the Modern Fauna became highly diverse in wider marine ecosystems only after the Paleozoic (*27*). However, we show that they were an important component of reef-supporting regions much earlier, during the Paleozoic, suggesting an important role for reefs as cradles of higher taxonomic composition throughout the evolutionary history of animals. As hotspots of diversity and potential net exporters of species to other marine environments, degradation of reefal habitats now and into the future (*34*–*39*) could therefore restructure marine ecosystems with major consequences for marine biodiversity over extended geological timescales.

## MATERIALS AND METHODS

### Occurrence data downloads

We reconstructed patterns of local- and regional-scale diversity for Phanerozoic marine invertebrates and major subgroups using occurrence data downloaded from the Paleobiology Database (*22*). Genus-level occurrence data for the taxon set Animalia excluding Chordata from marine environments were downloaded from the Paleobiology Database on 11 July 2024, using the PaleoDB Application Programming Interface URL “https://paleobiodb.org/data1.2/occs/list.csv?base_name=Animalia%5EChordata&idtype=latest&pres=regular&envtype=marine&idreso=lump_gensub&idqual=genus_certain&taxon_status=valid&show=attr,classext,subgenus,abund,ecospace,taphonomy,ctaph,etbasis,pres,coll,coords,loc,paleoloc,time,timebins,stratext,lithext,env,geo,methods,resgroup,ref,refattr,ent,entname,crmod”(also available in the supplemental code at 10.5281/zenodo.16098292). Subgenera were elevated to their respective genera. To visualize patterns within groups and to reconstruct patterns within Sepkoski’s three evolutionary faunas (*15*), we additionally defined taxon sets for major groups of marine animals, including Anthozoa, Bivalvia, Brachiopoda, Cephalopoda, Crinoidea, Echinoidea, Gastropoda, Porifera, and Trilobita, using downloads of PaleoDB occurrence numbers. Sepkoski’s evolutionary faunas comprise the Cambrian Fauna (Trilobita, Linguliformea, Graptolithina, and Conodonta); the Paleozoic Fauna (Anthozoa, Ostracoda, Rhynchonelliformea, Cephalopoda, and Crinoidea), and the Modern Fauna (Bryozoa, Bivalvia, Gastropoda, Echinoidea, and Chondrichthyes). Before filtering and binning, there were 800,928 occurrences.

### Occurrence data filtering criteria

Occurrence data were filtered following standard practices for removing unsuitable occurrences [e.g., (*8*, *10*)]. We excluded (i) uncertain identifications (i.e., retaining only valid taxa), (ii) collections with geographic scale listed as “basin” or stratigraphic scale listed as “group,” (iii) fossils preserving soft parts, (iv) coprolites and traces (i.e., retaining only body fossils with preservation mode given as “regular”), and (v) terrestrial and freshwater taxa.

To more fully control for biases arising from systematic changes in the mode of lithification of fossil-bearing sediments through the Phanerozoic, we restricted our focal analyses to collections that explicitly represent lithified deposits or poorly lithified deposits that have not been sieved (i.e., excluding unlithified and poorly lithified and sieved deposits). Lithification biases arise because unlithified and poorly lithified deposits are more prevalent in the fossil record from the Late Cretaceous through to the Recent [(*24*, *25*); fig. S11]. Counts of taxa from fossil deposits that are unlithified or poorly lithified are inflated relative to those from lithified sediments because they facilitate easier extraction of fossil specimens, especially those that are small (*24*, *40*–*42*). Past studies of Phanerozoic marine animal diversity [e.g., (*8*, *10*, *16*, *17*, *43*)] have excluded fossil data identified as explicitly originating from deposits that are either unlithified or poorly lithified and sieved. However, many collections in the PaleoDB lack information about lithification style (*n* = 53,516 or 37.2% of filtered data). Past studies typically have not excluded collections lacking lithification data, but our analyses (see below) suggest that many of these untagged collections likely derive from unlithified sediments. Because of this, it is not possible to fully control for lithification biases when including collections that lack information on lithification style.

Therefore, all of our focal analyses exclude unlithified or poorly lithified and sieved data, as per previous studies, but additionally exclude collections lacking information on lithification style. However, we also present results for multiple additional sifting criteria, including only excluding data that had been specifically tagged as being unlithified or poorly lithified and sieved, as per previous studies, and results are similar (fig. S10). Our full set of sifting criteria comprise the following: (i) all lithification styles, including collections lacking information on lithification; (ii) excluding collections representing both unlithified and poorly lithified-and-sieved deposits but retaining collections lacking information on lithification style [the default sifting criteria used by previous studies of Phanerozoic marine animal diversity; e.g., (*10*, *16*, *17*)]; (iii) excluding collections representing both unlithified and poorly lithified-and-sieved deposits and additionally excluding collections lacking information on lithification style; (iv) collections explicitly identified as representing lithified deposits; (v) unlithified or poorly lithified collections only; and (vi) collections lacking information on lithification style only.

After removing unsuitable occurrences and binning into approximately equal-length time bins (see below; table S7), the data comprised 143,773 collections containing 699,642 occurrences, representing 30,209 genera (*22*).

### Time binning of occurrence data

Occurrence data were binned into approximately equal-length time bins to control for the high variability in geological stage durations, especially toward the present-day [table S7; this practice follows past studies, such as (*8*, *10*)]. The filtered and binned occurrence data comprised 143,773 collections containing 699,642 occurrences, representing 30,209 genera (*22*). We also binned data into geological stages to demonstrate the effect of different binning schemes, which do not change overall patterns (see fig. S23).

### Spatial standardization

Paleocoordinates for fossil collections were rotated to the map age closest to the midpoint of each time bin using the R package chronosphere [version 0.6.1, online reconstruction method; (*44*)] with the PALEOMAP paleogeographic model of Scotese (*45*) (we also conducted sensitivity analyses using alternative plate models; see the “Effect of plate model choice” section). We standardized the extent of spatial sampling by binning fossil localities into equal-area hexagonal/pentagonal grid cells with 100-, 500-, and 1000-km spacings (Fig. 1) using the dggridR R package version 2.0.3 (*46*) (see Materials and Methods). These grid cell spacings were chosen to balance regional extent versus the need for sufficient sampling and are comparable to regional spatial samples used in our previous work (*10*).

### Calculations of regional diversity and other variables

For each grid cell, we calculated sampling-standardized genus richness using SQS [(*8*, *16*), also known as coverage-based rarefaction (*23*, *47*)], at a quorum level (target sample coverage) of 0.9, using the R package iNEXT version 2.0.20 (*48*), which allows both drawing down samples (subsampling or interpolating) and extrapolating to equal coverage. Following (*23*), we discarded extrapolated estimates based on extrapolated samples that were more than twice the reference sample size.

We also calculated a range of other variables for each grid cell, including the Berger-Parker dominance index (*49*) as a measure of evenness, face-value (raw or unstandardized) counts of genera, counts of fossil collections (the terminology used in the Paleobiology Database for samples of taxa from fossil localities that are well-circumscribed in time and space), counts of fossil occurrences (i.e., the confirmed presence of a taxon in a fossil collection), counts of references (i.e., publications associated with fossil occurrences), counts of geological formations, and counts of distinct paleocoordinate locations.

### Quality criteria for excluding poorly sampled grid cells

We excluded cells that did not meet certain data quality criteria, indicating minimal sampling (our “quality criteria”). Cells were excluded if they contained fewer than 10 collections or two references. This process was intended to eliminate grid cells that have spuriously high levels of sample completeness [measured by Good’s *u* (*50*)] resulting from estimation error due to small sample sizes (i.e., poorly sampled grid cells that spuriously appeared to have high sample coverage by virtue of there being no or few singletons, despite very limited sampling—for example, if a cell contained a small number of spatially clustered collections that had all yielded similar taxon lists; in this case, Good’s *u* would be high despite sampling within the cell being very limited). More generally, however, per-cell SQS diversity estimates scale quite linearly with counts of collections (fig. S24), references (fig. S25), and distinct paleocoordinates (fig. S26). It is likely that these linear scaling relationships represent a difficult-to-disentangle combination of stochastic variation in sampling intensity (with some cells being exceptionally well-sampled and others being more poorly sampled) and genuine variation in richness driving variation in counts of these sampling proxies (e.g., higher true richness in a region sometimes encouraging more intensive collection and thus pushing up counts of collections, references, and distinct paleocoordinates). For this reason, we have adopted quality criterion filtering quotas that aim to weed out only the worst-sampled cells. However, for comparative purposes, we show the effect of imposing a wide range of minimum quotas for counts of collections (fig. S27), references (fig. S28), and counts of distinct paleocoordinate locations (fig. S29). We also show the effect of our overall quality criteria (at least 10 collections and two references) versus retaining all data (fig. S30). The results are largely congruent regardless of the choice of quality criterion thresholds.

### Effect of plate model choice

Our focal results use the PALEOMAP plate model (*51*) for reconstructing the paleocoordinates of fossil localities. However, there is considerable variation in estimates for different plate tectonic rotation models (*52*). We therefore also performed our complete analyses using the alternative plate models: “MERDITH2021” (*53*), “MULLER2022” (*54*), and “GOLONKA” (*55*). Differences in paleolatitudinal distributions of reef-supporting and non–reef-supporting grid cells are shown in fig. 35. However, differences in latitudinal distributions of cells have no bearing on patterns of regional-scale diversity through time, which are visually identical between the focal and alternate plate models (because of this, results for other plate models are not shown).

Some high-paleolatitude (≥75° paleolatitude) reef-supporting cells appear in the PALEOMAP reconstruction. These cells do appear to be reefal (containing the Nemo Formation of New Zealand in the Changhsingian, lithology “reef rocks” and environment “reef, buildup, or bioherm,” and the Khabt-el-Hajar Formation of Morocco in the Late Ordovician (Ashgill), lithology “limestone” and environment reef, buildup or bioherm). Their high paleolatitude could either be genuine or due to errors in the PALEOMAP model.

### Local and formation scale richness

To analyze patterns of genus richness at the local scale, we tallied face-value counts of genera per collection and per formation. Face-value counts at these spatial scales are heavily right-skewed [i.e., most collections contain only one or a small handful of taxa, but some collections are extraordinarily diverse and represent a fairly complete snapshot of local-scale diversity (*4*, *56*)]. To exclude uninformative collections and formations, we imposed quality criteria indicative of sampling level, based on counts of references, collections, and higher taxa (defined here as Mollusca, Arthropoda, Echinodermata, Cephalopoda, Anthozoa, Chordata *sans* Tetrapoda, Bryozoa, Porifera, Graptolithina, and Annelida): We retained collections with at least five references per collection or four higher taxa and formations with at least 20 collections, five references, or four higher taxa (note that this is a different quality criterion procedure to that discussed above for regional-scale analyses using grid cells; different quality criteria are needed for analyses using per-collection and per-formation counts of genera versus diversity estimates for grid cells).

### Classification of reef-supporting and non–reef-supporting grid cells

We classified cells as reef-supporting if they contained at least one fossil collection that represented reefal facies in the Paleobiology Database occurrence download. As for past studies [e.g., (*8*)], reefal environmental facies were identified using the “environment” field and the values “reef, buildup, or bioherm,” “slope/ramp reef,” “perireef or subreef,” “basin reef,” “platform/shelf-margin reef,” and “intrashelf/intraplatform reef.” Non-reefal facies comprised all other terms (“offshore,” “peritidal,” “carbonate indet.,” “offshore shelf,” “shallow subtidal indet.,” “lagoonal,” “sand shoal,” “transition zone/lower shoreface,” “deep subtidal shelf,” “offshore ramp,” “submarine fan,” “prodelta,” “shoreface,” “offshore indet.,” “deep subtidal indet.,” “open shallow subtidal,” “deltaic indet.,” “deep subtidal ramp,” “foreshore,” “interdistributary bay,” “delta plain,” “estuary/bay,” “coastal indet.,” “delta front,” “lagoonal/restricted shallow subtidal,” “paralic indet.,” and “deep-water indet.”). If only imprecise environmental types were present in a cell (e.g., “marine indet.”), then that grid cell was not assigned to either reef-supporting or non–reef-supporting and was discarded.

We considered the presence of a single reefal collection sufficient to show that the geographic region encompassed by the cell was conducive to supporting reefal ecosystems. However, classifying a grid cell as non–reef-supporting when it actually supported reefs (i.e., a “false negative”) is likely to be a more important source of error, due to cells that did support reefal ecosystems in deep time but which have not yielded reefal facies due to their restricted spatial distribution and thus lower likelihood of preservation. There is a linear increase in the log of SQS diversity with the number of reefal collections present in a cell (fig. S2B). There is also a strong linear relationship between the log of SQS diversity and the total number of collections present in a cell for both reef-supporting and non–reef-supporting regions, albeit with reef-supporting regions showing a steeper relationship than non–reef-supporting regions (fig. S2A). This steeper slope in reef-supporting cells may indicate higher among-collection β diversity in these environments. We did not assign cells to reef-supporting or non–reef-supporting categories based on the proportion of reefal cells (as opposed to counts), because the number of total collections present in a cell declines as the proportion of reefal collections increases (fig. S2D), and this causes the diversity of cells to decrease as the proportion of reefal collections increases (fig. S2C).

It is possible that some “carbonate indet.” facies indicate reefal environments, but it is difficult to know which these are. Within carbonate facies, however, the difference in diversity between reef-supporting and non–reef-supporting regions is much more pronounced than within siliciclastic lithologies (fig. S12). This suggests that any carbonate facies that have not been associated with reefs are likely to be genuinely less diverse than those that do support reefs, so misassignment is unlikely to be problematic.

We compared our assignments of reef-supporting and non–reef-supporting status based on PaleoDB environment data to those derived from the PARED PaleoReefs database (*57*), based on palaeocoordinates of recorded reef sites within grid cells, and found strong agreement (i.e., assignments to reef-supporting or non–reef-supporting categories are the same using PBDB and PARED data for the vast majority of cases and only differ in a small minority of cases; see fig. S3). Therefore, we considered the environmental information in the Paleobiology Database to be sufficient to identify reef-supporting regions.

For Fig. 4, which parses reef-supporting and non–reef-supporting patterns according to the types of reef-building organisms present in each cell, reef-supporting cells were identified as being associated with particular types of reef-building organisms using the palaeocoordinates of reef sites listed in the PARED PaleoReefs database (*57*). Note that grid cells can appear in more than one panel if they contain more than one type of reef-building organism.

### Statistical tests

To test for statistically significant differences in diversity among groups (e.g., between reef-supporting and non–reef-supporting regions during the Pre- and Post-K/Pg time intervals and within reef-supporting and non–reef-supporting regions across the pre- and post-K/Pg intervals), we conducted nonparametric Wilcoxon tests using the function wilcox_test() in the R package “rstatix” (version 0.7.0). To quantify effect sizes for differences between groups in Wilcoxon tests, we also computed the rank-biserial correlation (“*r* value”) using the function wilcox_effsize() in the R package “rstatix,” which is more appropriate than Cohen’s *d* (*58*) for this nonparametric test.

### Rarefaction analyses

To investigate the effects of more intense sampling in some grid cells, especially those from Europe and North America, we rarefied data within grid cells by a range of variables that may represent proxies for sampling intensity, including collections, references, and distinct paleocoordinate locations. Diversity often increases linearly with counts of these variables (figs. S24 to S26). Data for reef-supporting and non–reef-supporting regions were rarefied (1000 subsampling trials) using these variables at a range of quotas (see figs. S32 to S34) and visualized both for global data and within modern continental regions. For reasons of computational efficiency, face-value counts of genera were used.
