# Supplementary material for: Reefal regions were biodiversity hotspots throughout the Phanerozoic
Source: Sci Adv. 2025 Nov 5;11(45):eadv9793. doi: 10.1126/sciadv.adv9793 (PMC12588287; doi:10.1126/sciadv.adv9793)
Supplement: Supplementary file 1 — Figs. S1 to S34 Tables S1 to S7 [file sciadv.adv9793_sm.pdf]

Science Advances
AAAS

Supplementary Materials for

Reefal regions were biodiversity hotspots throughout the Phanerozoic

Roger A. Close *et al.*

Corresponding author: Roger A. Close, roger.close@earth.ox.ac.uk

Sci. Adv. **11**, eadv9793 (2025)
DOI: 10.1126/sciadv.adv9793

This PDF file includes:

Figs. S1 to S34
Tables S1 to S7

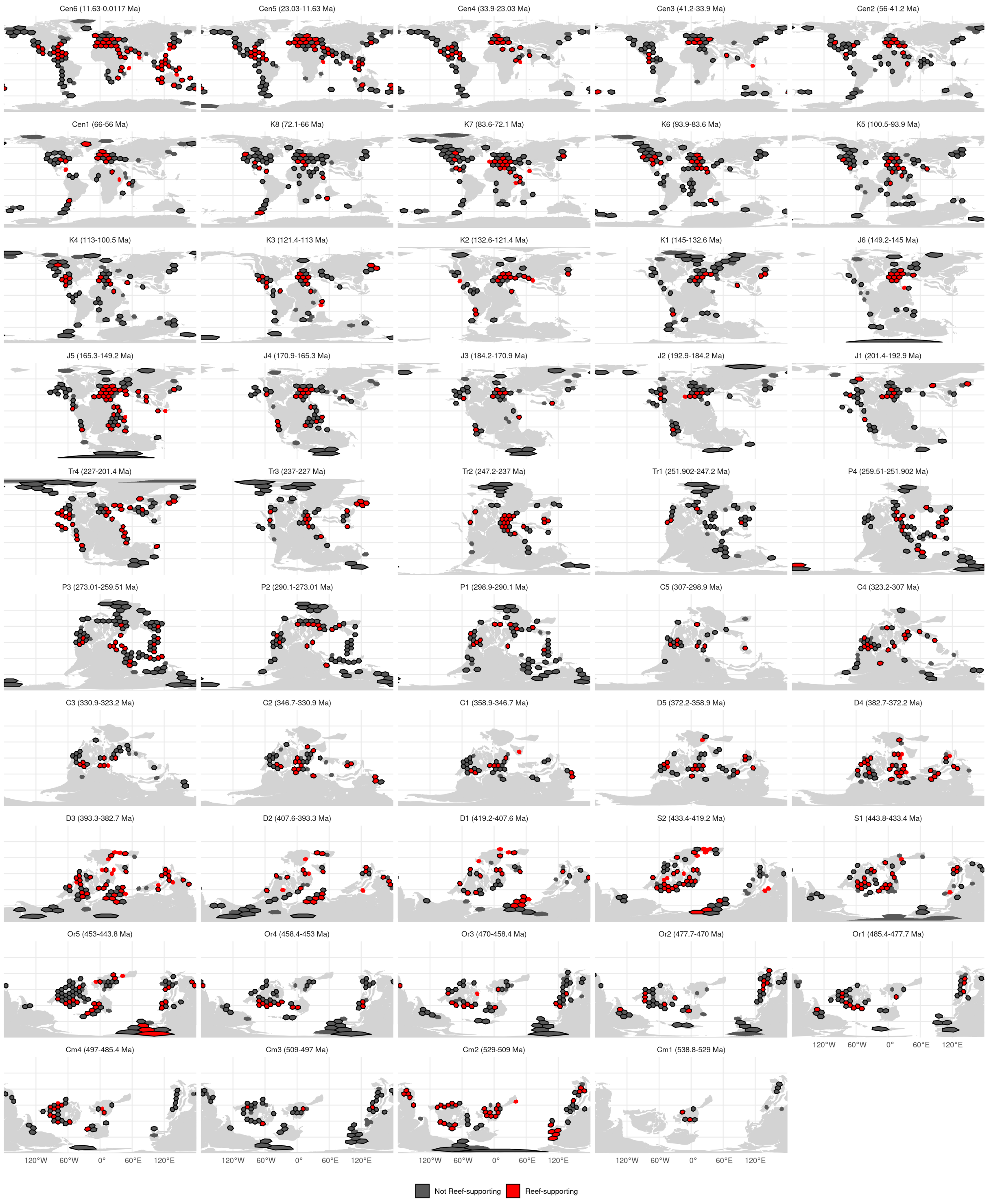

Fig. S1: Paleogeographic distributions of reef-supporting (red) and non-reef-supporting (gray) regions (equal-area hexagonal/pentagonal grid cells with 1000 km spacings) for all of the equal-length time intervals analyzed (table S7). Black borders denote grid cells that meet our quality criteria, while those without black borders are those that do not. Paleomaps from the PALEOMAP project (*45*), with plate reconstructions via rgplates R package (CC BY 4.0 license; https://creativecommons.org/licenses/by/4.0/).

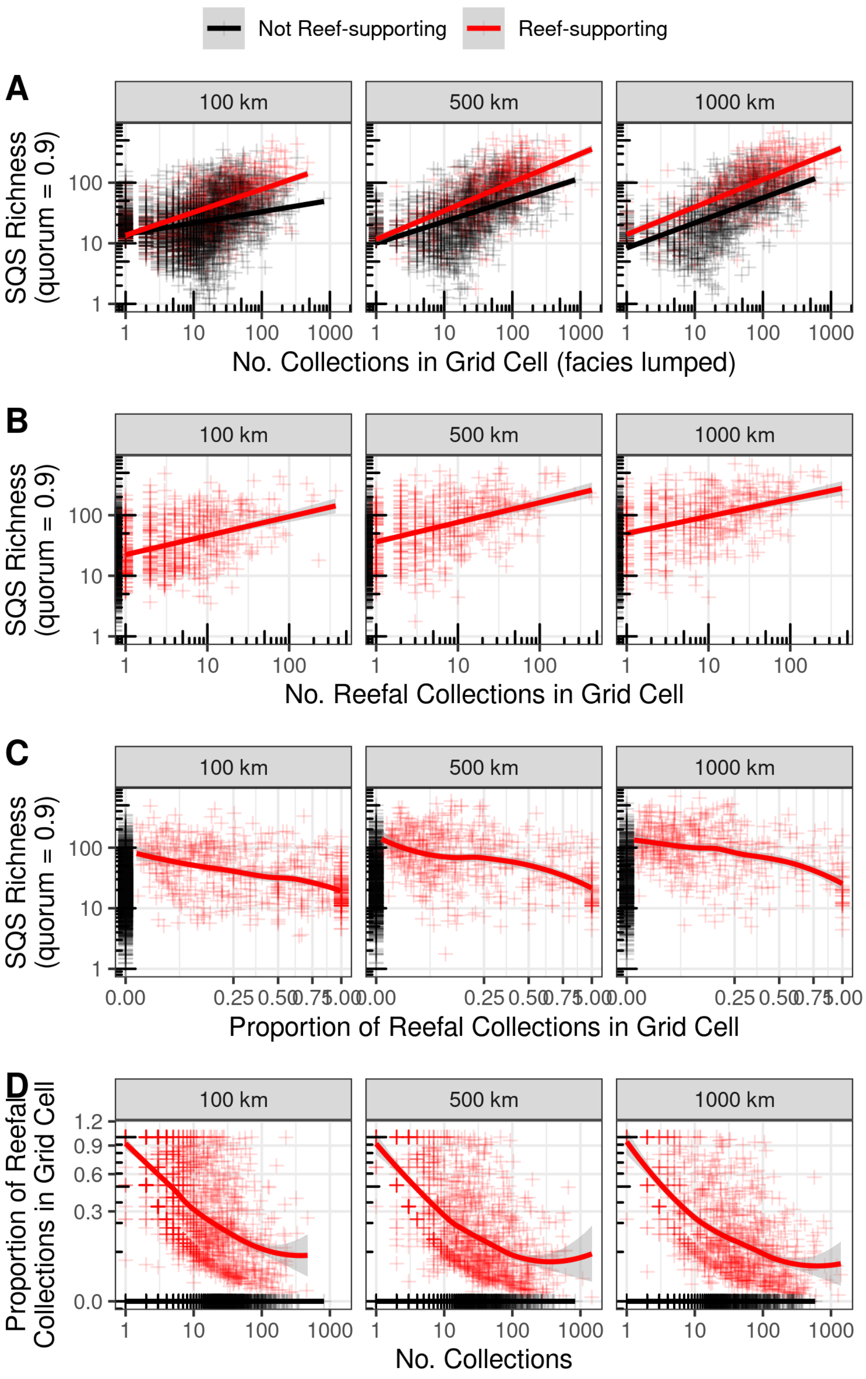

Fig. S2: **Relationships between collection counts, SQS diversity, and proportions of reefal collections.** Relationships between (A) diversity (SQS, quorum = 0.9) and the total number of collections of any facies present in a region; (B) diversity (SQS, quorum = 0.9) and the number of reefal-type collections present in a region; (C) diversity (SQS, quorum = 0.9) and the proportion of reefal-type collections (versus non-reefal) present in a region; and (D) the proportion of reefal-type collections (versus non-reefal) and counts of collections present in a region, all for reef-supporting (red) and non-reef-supporting (black) regions. Regions represent equal-area hexagonal/pentagonal grid cells with 100 km, 500 km, 1000 km and 2000 km spacings. Lines represent OLS linear model fits in panels A and B and LOESS fits in panels C and D. Counts of collections and SQS diversity have log-transformed axes, while proportions of reefal collections are square-root–transformed. Crosses represent SQS diversity estimates for individual grid cell regions. PaleoDB collection data excludes those identified as unlithified and poorly-lithified-and-sieved deposits, but includes collections with no information on lithification style. Non-reef-supporting data points are confined to the left-hand side of the plot in panels B and C, and to the bottom of the plot in panel D, since they lack any reefal collections. Panel (C) shows that diversity declines as the proportion of reefal collections increases. This is because regions containing a high proportion of reefal collections also tend to have a smaller absolute number of collections, as shown in panel D.

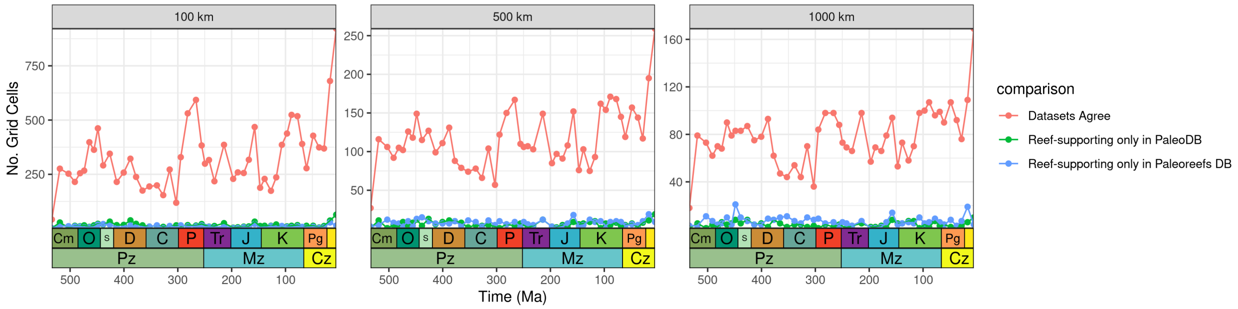

Fig. S3: Agreement between assignments of reef-supporting and non-reef-supporting status for equal-area grid cells (equal-area hexagonal/pentagonal grid cells with 100 km, 500 km and 1000 km spacings) using PARED PaleoReefs database (*57*) and presence of reefal facies in the Paleobiology Database (*22*).

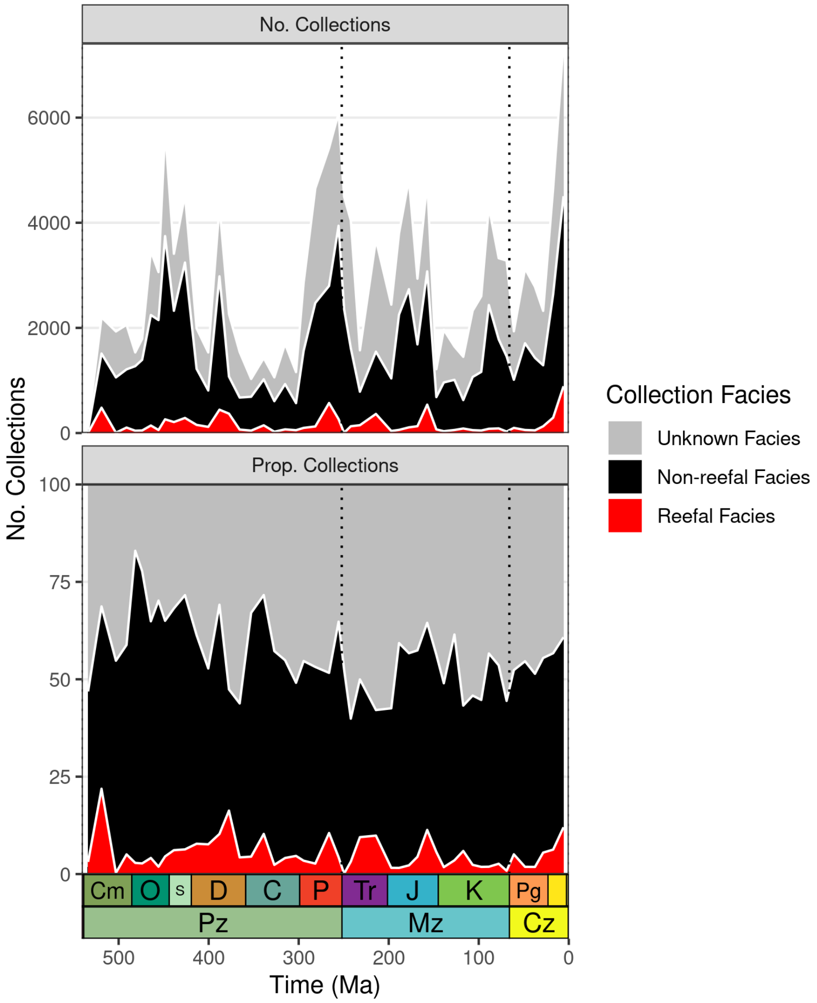

Fig. S4: Counts of collections representing either reefal facies, non-reefal facies, or unknown facies (i.e., PaleoDB collections lacking information on environmental facies) through the Phanerozoic, using equal-length time bins.

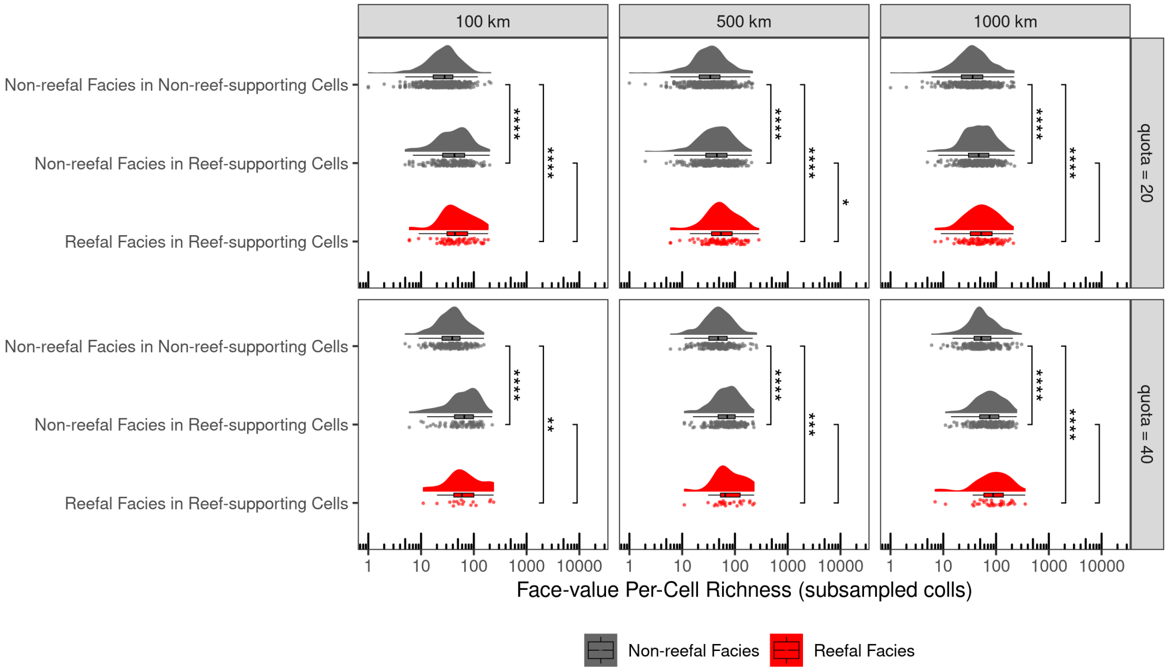

Fig. S5: Distributions of diversity for Phanerozoic reef-supporting and non-reef-supporting regions (defined using equal-area grid cells of 100 km, 500 km and 1000 km) after subsampling to equal counts of reefal (red) or non-reefal (gray) collections at quotas of 20 and 40. These results show that, when controlling for variation in sampling intensity within cells, reef-supporting cells host higher levels of diversity than non-reef-supporting cells, regardless of whether reefal facies or non-reefal facies are analyzed. Each plot shows three ways of visualizing the data: individual data points with jitter, a boxplot (line denotes the median value, the hinges of the box correspond to the interquartile range, and whiskers extend from the hinges to the smallest/largest values at most 1.5 * IQR of the hinge), and a density plot using a Gaussian kernel with a smoothing bandwidth of 1. Statistical significance for Wilcoxon tests between groups is indicated by either no text ($p > 0.05$), * ($p \leq 0.05$), ** ($p \leq 0.01$), *** ($p \leq 0.001$) or **** ($p \leq 0.0001$).

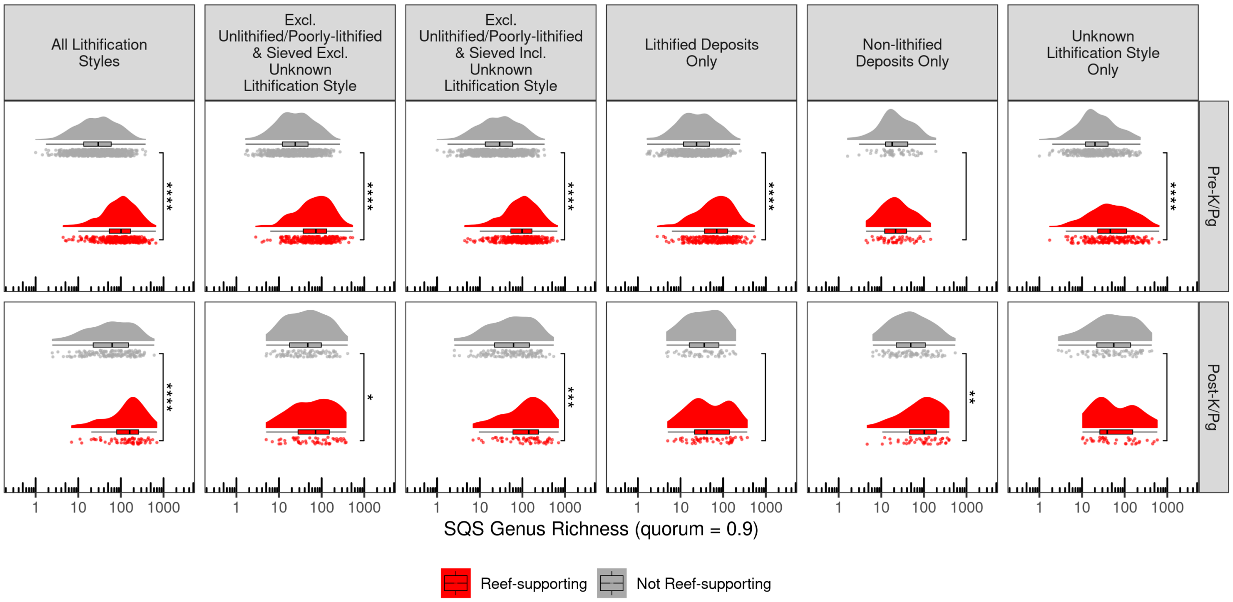

Fig. S6: Distribution of diversity within reef-supporting (red) and non-reef-supporting (gray) regions (equal-area hexagonal/pentagonal grid cells with 1000 km spacings) in the Paleozoic–Mesozoic and Cenozoic. Each plot shows three ways of visualizing the data: individual data points with jitter, a boxplot (line denotes the median value, the hinges of the box correspond to the interquartile range, and whiskers extend from the hinges to the smallest/largest values at most 1.5 * IQR of the hinge), and a density plot using a Gaussian kernel with a smoothing bandwidth of 1. Statistical significance for Wilcoxon tests between groups is indicated by either no text ($p > 0.05$), * ($p \leq 0.05$), ** ($p \leq 0.01$), *** ($p \leq 0.001$) or **** ($p \leq 0.0001$).

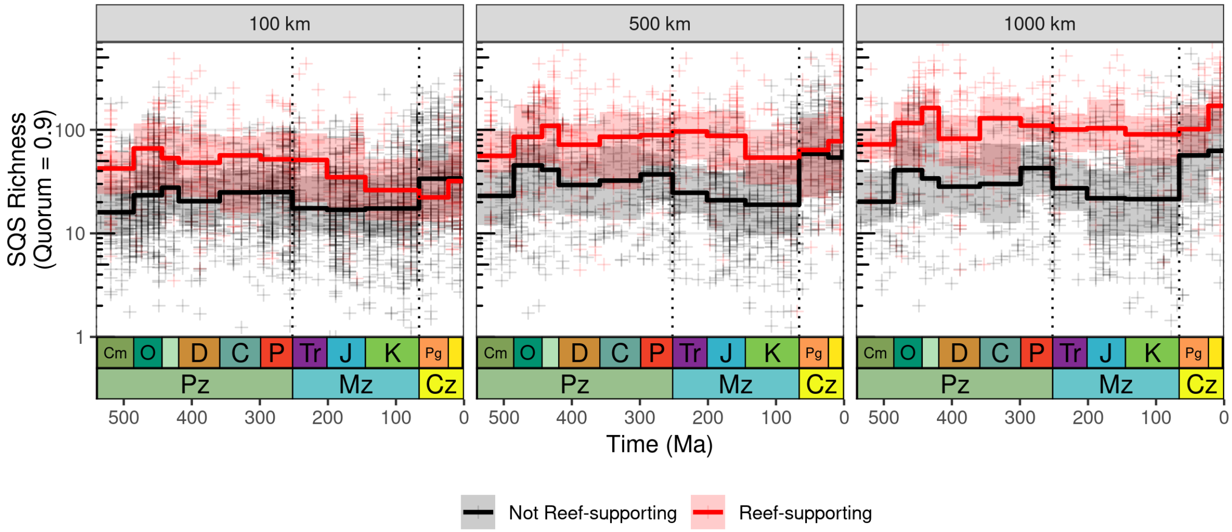

Fig. S7: Patterns among reef-supporting and non-reef-supporting regions for all grid-cell sizes (spacings of 100 km, 500 km and 1000 km). Crosses represent SQS diversity estimates for individual grid cell regions, while trend lines represent medians and interquartile ranges of regional diversity for geological periods.

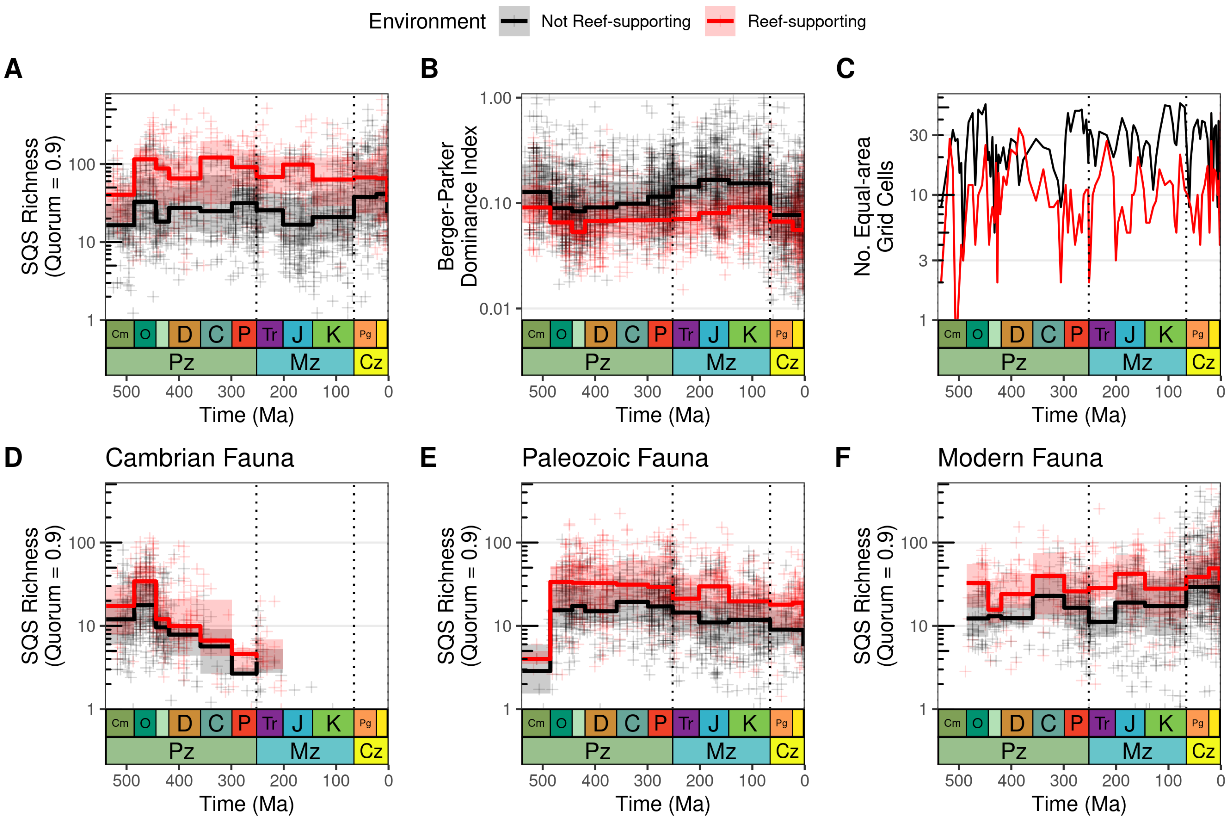

Fig. S8: As for main figure, but using stage-length time bins for analysis rather than equal-length time bins. Summary trend lines are at the scale of geological periods. Differences in Phanerozoic marine invertebrate animal diversity patterns between reef-supporting (red) and non-reef-supporting (black) regions (equal-area hexagonal/pentagonal grid cells with spacings of 1000 km for all panels in this figure), excluding collections explicitly identified as representing unlithified or poorly-lithified-and-sieved deposits, but retaining collections that have no information about lithification style. Dotted lines represent boundaries between geological eras. Note logarithmic y-axes. For panels A–B and D–F, crosses represent SQS diversity estimates for individual grid cell regions, while trend lines represent medians and interquartile ranges of regional diversity for stage-length bins. (A) Spatially-standardized Phanerozoic marine animal diversity, contrasting patterns for reef-supporting and non-reef-supporting regions. Note that in reef-supporting regions, levels of diversity have been broadly similar since the Ordovician, with no evidence for long-term, secular trends. In non-reef-supporting regions, by contrast, levels of diversity were similar from the Ordovician to the latest Cretaceous, when diversity rose fairly rapidly to a new, higher level that was sustained through the Cenozoic. However, this K/Pg increase is strongly associated with gastropods and unlithified sediments (see fig. S9). (B) Evenness, estimated using Berger-Parker dominance index (*49*), in reef-supporting and non-reef-supporting grid cells. (C) Counts of reef-supporting and non-reef-supporting cells through the Phanerozoic, using equal-length time bins. Panels (D–F) show patterns for Sepkoski's evolutionary faunas. (D) Cambrian Fauna (Trilobita, Linguliformea, Graptolithina, Conodonta); (E) Paleozoic Fauna (Anthozoa, Ostracoda, Rhynchonelliformea, Cephalopoda, Crinoidea); (F) Modern Fauna (Bryozoa, Bivalvia, Gastropoda, Echinoidea, Chondrichthyes).

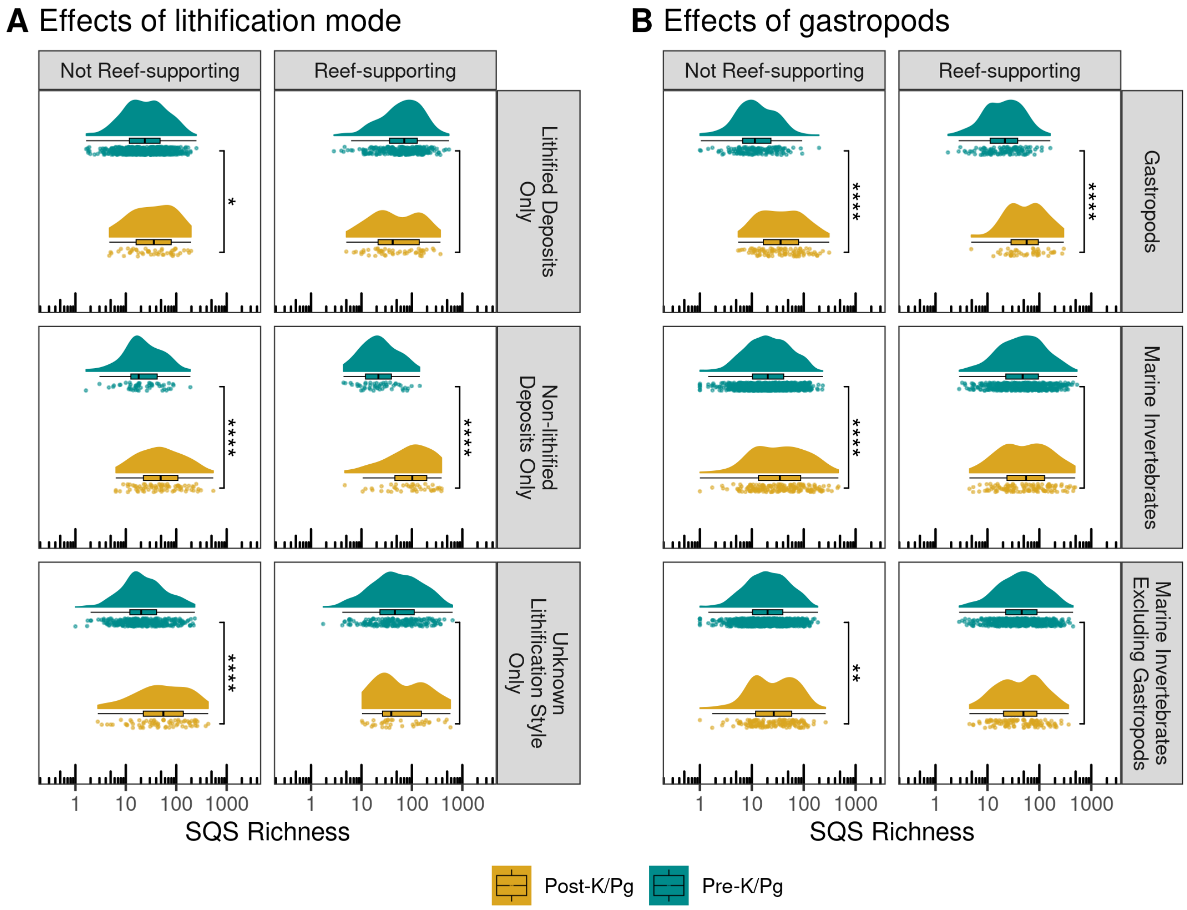

Fig. S9: Potential drivers of the apparent increase in diversity of non-reef-supporting regions that occurred at or just before the K/Pg. (A) Effects of lithification style on diversity within reef-supporting and non-reef-supporting regions across the K/Pg boundary. (B) Effects of including or excluding gastropods on diversity in marine animals within reef-supporting and non-reef-supporting regions across the K/Pg boundary. Gastropods experienced a large increase in diversity in the latest Cretaceous, especially in non-reef-supporting regions. When gastropods are excluded from diversity estimates for marine invertebrates, little increase across the K/Pg is evident, suggesting that this event is primarily driven by the diversity dynamics of gastropods. Each plot shows three ways of visualizing the data: individual data points with jitter, a boxplot (line denotes the median value, the hinges of the box correspond to the interquartile range, and whiskers extend from the hinges to the smallest/largest values at most 1.5 * IQR of the hinge), and a density plot using a Gaussian kernel with a smoothing bandwidth of 1. Statistical significance for Wilcoxon tests between groups is indicated by either no text ($p > 0.05$), * ($p \leq 0.05$), ** ($p \leq 0.01$), *** ($p \leq 0.001$) or **** ($p \leq 0.0001$).

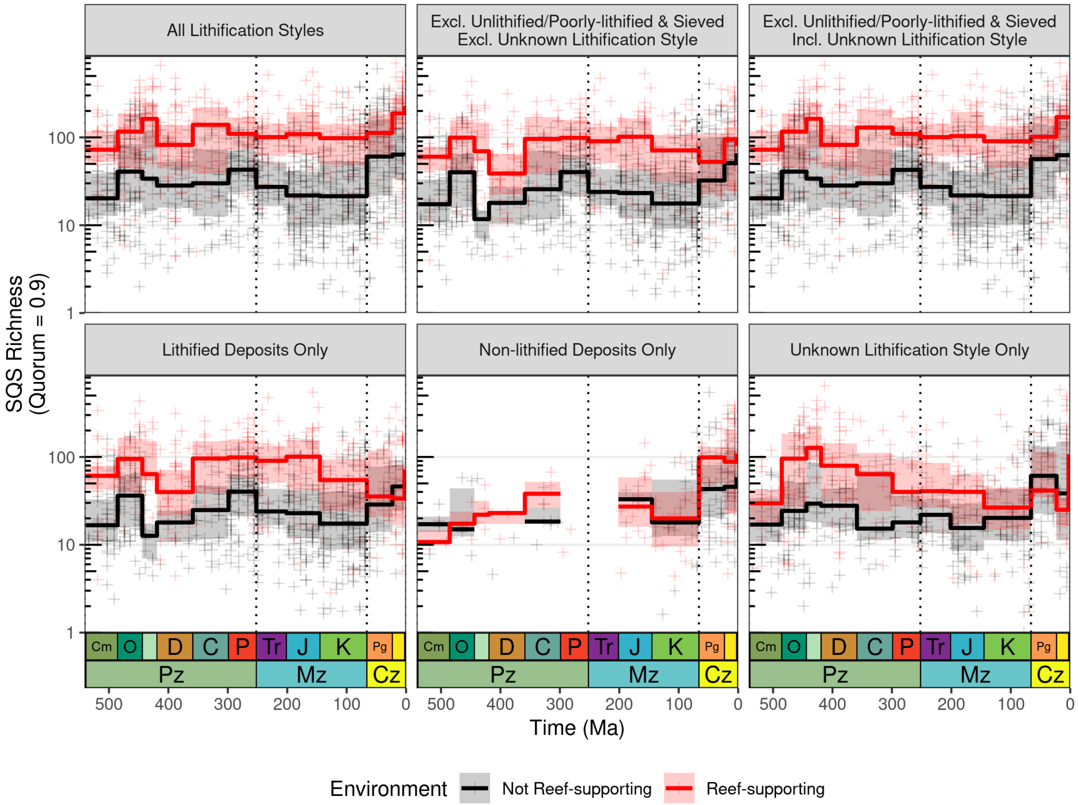

Fig. S10: Differences in Phanerozoic marine invertebrate animal diversity patterns between reef-supporting (red) and non-reef-supporting (black) regions (equal-area hexagonal/pentagonal grid cells with spacings of 1000 km for all panels in this figure), comparing the effects of sifting criteria that vary the inclusion or exclusion of data associated with different lithification styles. See Supplementary Methods for discussion of these sifting criteria. Crosses represent SQS diversity estimates for individual grid cell regions, while trend lines represent medians and interquartile ranges of regional diversity for geological periods.

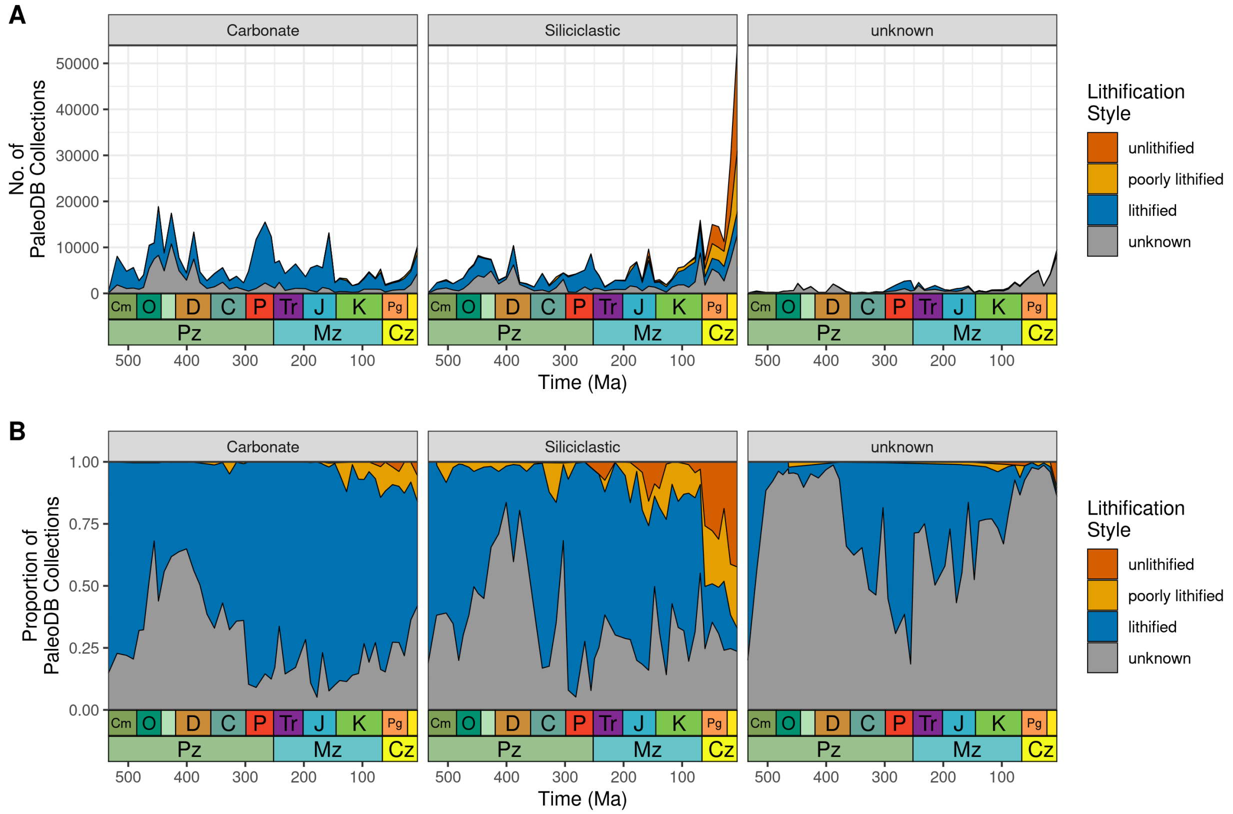

Fig. S11: Trends in lithification style through the Phanerozoic by lithology (siliciclastic, carbonate or unknown), showing (A) absolute counts of fossil collections ascribed to each lithification style (unlithified, poorly-lithified, or lithified), or (B) proportions of these counts.

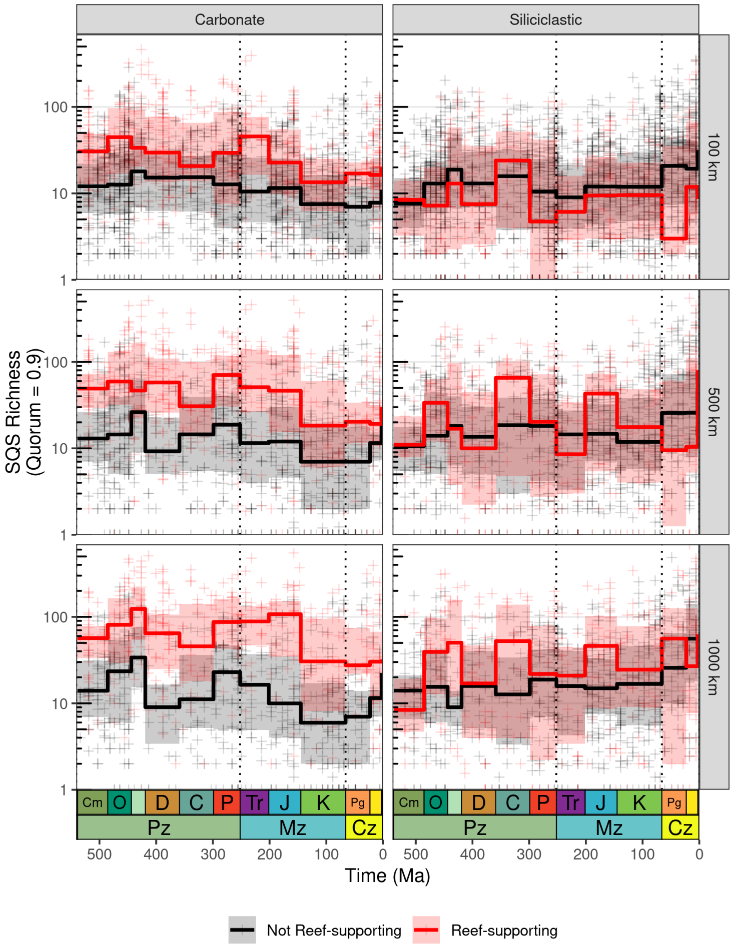

Fig. S12: The effect of splitting data into carbonate and siliciclastic lithologies on patterns among reef-supporting and non-reef-supporting regions for all grid-cell sizes (spacings of 100 km, 500 km and 1000 km). Crosses represent SQS diversity estimates for individual grid cell regions, while trend lines represent medians and interquartile ranges of regional diversity for geological periods.

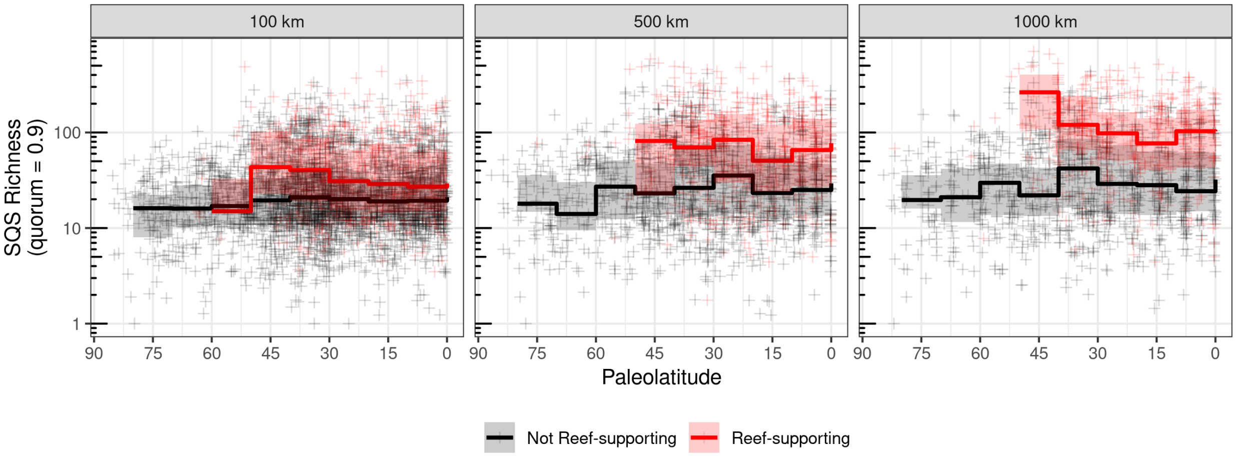

Fig. S13: Latitudinal gradients of Phanerozoic marine invertebrate animal diversity, separately showing trends across paleolatitudes for reef-supporting (red) and non-reef-supporting (black) regions (equal-area hexagonal/pentagonal grid cells with spacings of 100 km, 500 km, 1000 km and 2000 km). Collections explicitly identified as representing unlithified or poorly-lithified-and-sieved deposits were excluded. Note logarithmic y-axis. Crosses represent SQS diversity estimates for individual grid cell regions, while trend lines represent medians and interquartile ranges of regional diversity for 10 degree paleolatitudinal bins. Diversity is consistently higher in reef-supporting regions than in non-reef-supporting regions, even at low paleolatitudes, indicating that this effect is not solely driven by higher diversity in all tropical low-paleolatitude regions.

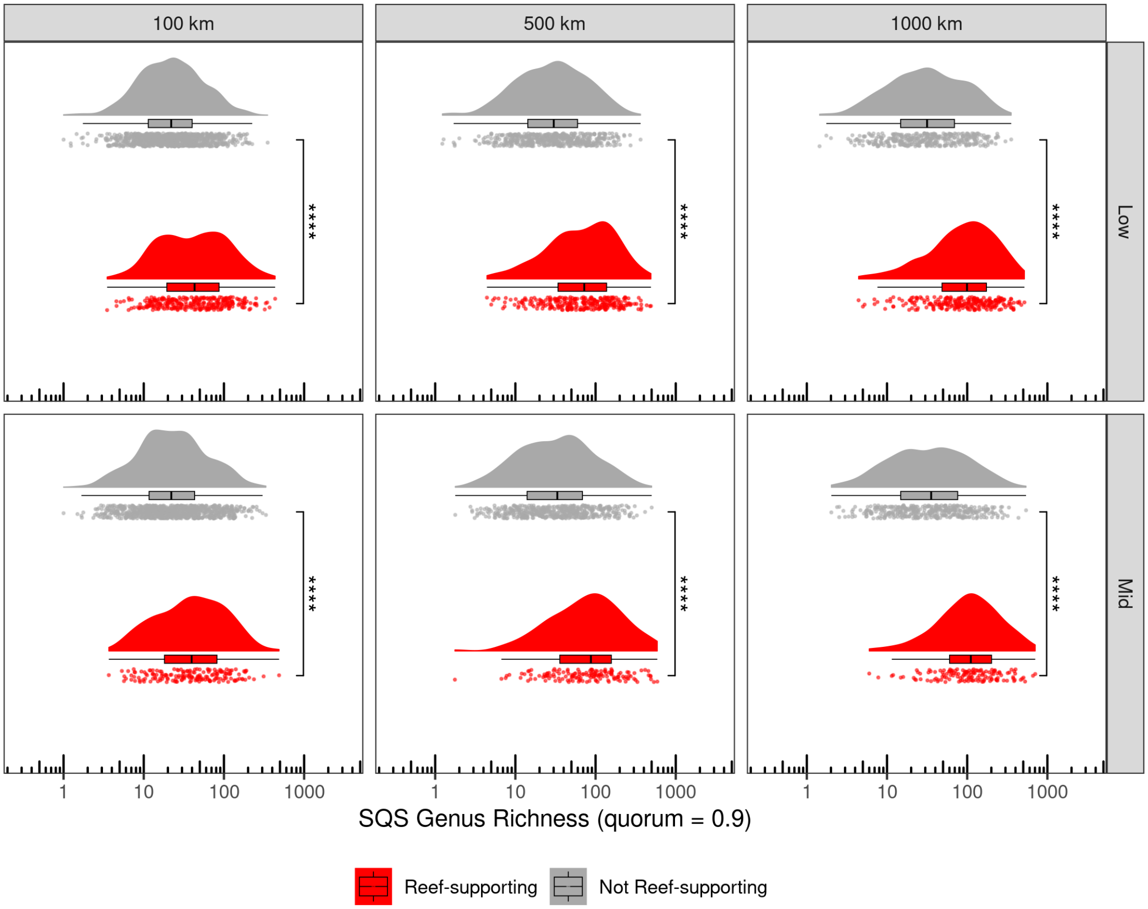

Fig. S14: Distributions of SQS diversity (quorum = 0.9) within reef-supporting (red) and non-reef-supporting (gray) regions (equal-area hexagonal/pentagonal grid cells with 100 km, 500 km, 1000 km and 2000 km spacings) between low (0–30°) and mid (30–60°) absolute paleolatitude zones. Diversity is consistently higher in reef-supporting regions than in non-reef-supporting regions, regardless of paleolatitude zones. Each plot shows three ways of visualizing the data: individual data points with jitter, a boxplot (line denotes the median value, the hinges of the box correspond to the interquartile range, and whiskers extend from the hinges to the smallest/largest values at most 1.5 * IQR of the hinge), and a density plot using a Gaussian kernel with a smoothing bandwidth of 1. Statistical significance for Wilcoxon tests between groups is indicated by either no text ($p > 0.05$), * ($p \leq 0.05$), ** ($p \leq 0.01$), *** ($p \leq 0.001$) or **** ($p \leq 0.0001$).

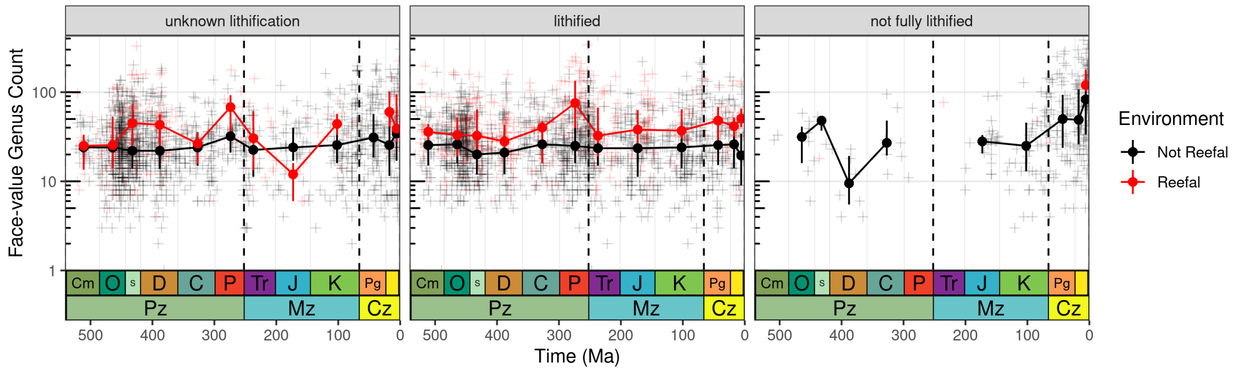

Fig. S15: Per-formation counts of genera for reef-supporting and non-reef-supporting geological formations, broken down by lithification style. To simplify the presentation of the data, panels show data of unknown lithification only, lithified data only, and data that is not fully lithified only, including unlithified and poorly-lithified deposits. Crosses represent counts of genera for individual formations, while trend-lines indicate medians and interquartile ranges for geological periods. Quality criteria were used to exclude poorly-sampled formations (at least 20 collections, five references or four higher taxa).

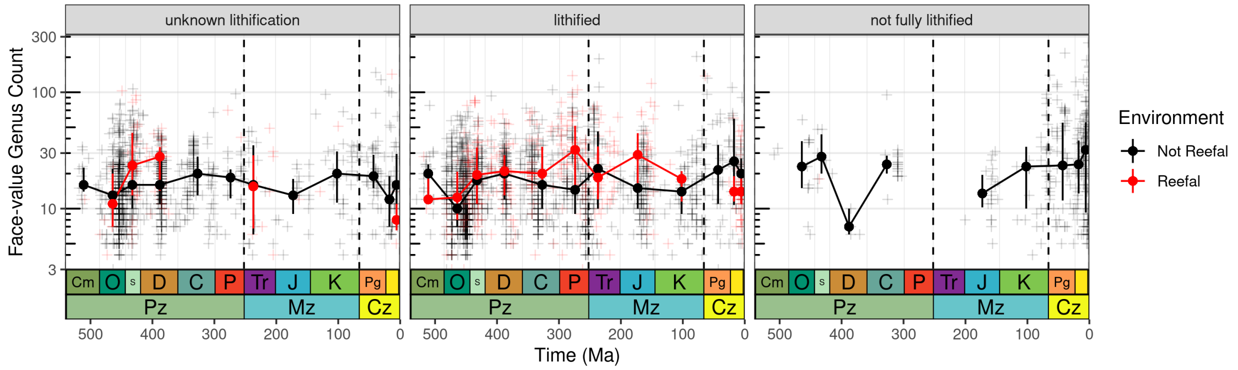

Fig. S16: Per-collection counts of genera for reefal and non-reefal facies, broken down by lithification style. To simplify the presentation of the data, panels show data of unknown lithification only, lithified data only, and data that is not fully lithified only, including unlithified and poorly-lithified deposits. Crosses represent counts of genera for individual collections, while trend-lines indicate medians and interquartile ranges for geological periods. Quality criteria were used to exclude poorly-sampled collections (at least five references per collection or four higher taxa).

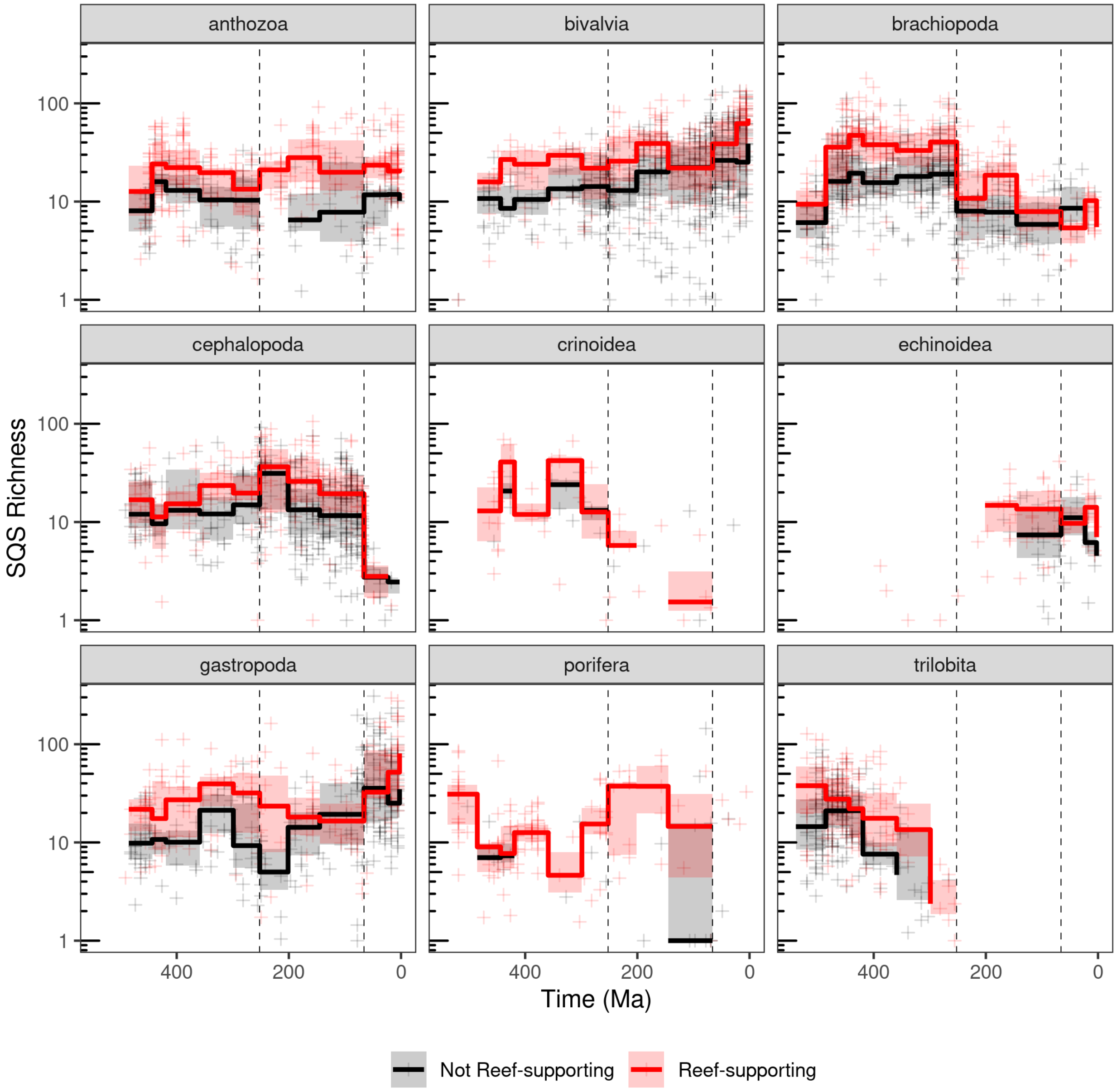

Fig. S17: Differences in diversity patterns for major clades of marine invertebrate animals between reef-supporting (red) and non-reef-supporting (black) regions (equal-area hexagonal/pentagonal grid cells with 1000 km spacings). Note logarithmic y-axes. Crosses represent SQS diversity estimates for individual grid cell regions, while trend lines represent medians and interquartile ranges of regional diversity for geological periods. Dashed lines represent boundaries between geological eras. PaleoDB collection data excludes those identified as unlithified and poorly-lithified-and-sieved deposits, but includes collections with no information on lithification style.

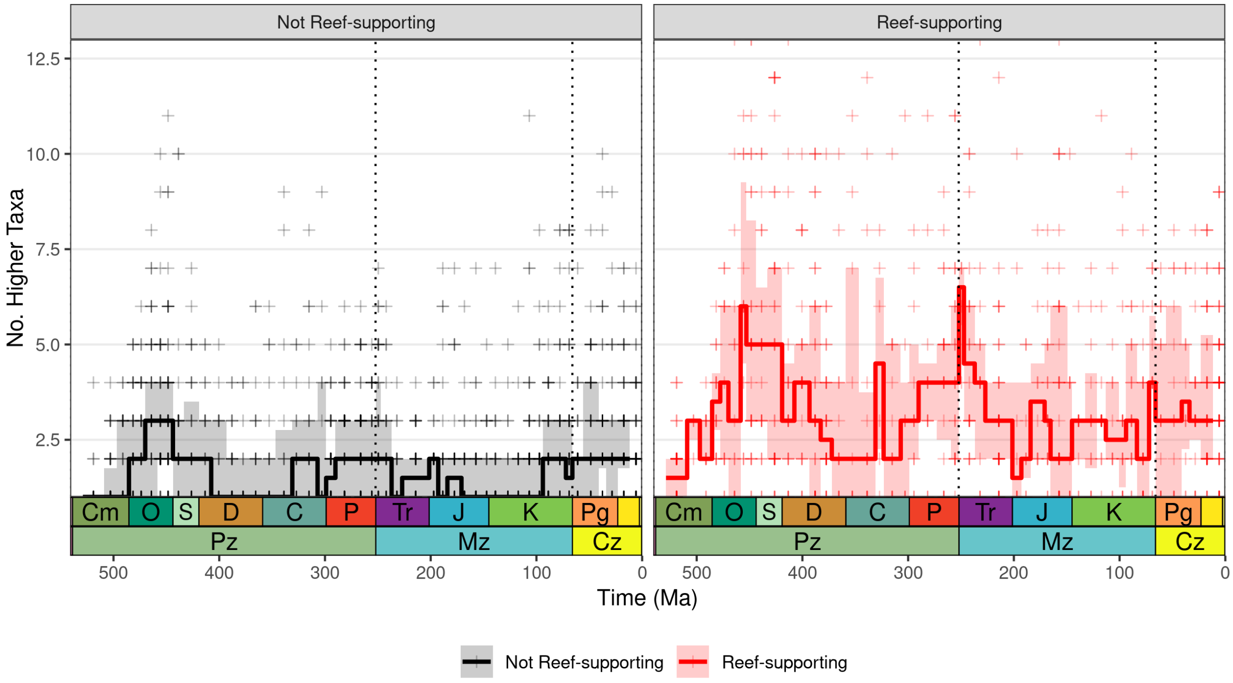

Fig. S18: Counts of higher taxa (major groups of taxa comprising Bivalvia, Rhynchonelliformea, Linguliformea, Cephalopoda, Gastropoda, Chordata *sans* Tetrapoda, Anthozoa, Trilobita, Conodonta, Bryozoa, Porifera, Tetrapoda, Crinoidea, Echinoidea, Graptolithina, Decapoda, Annelida) for reef-supporting (red) and non-reef-supporting (black) regions (1000 km equal-area hexagonal grid cells). Crosses represent face-value counts of these higher taxa for grid cells, and lines with transparent ribbons represent medians and interquartile ranges for geological periods.

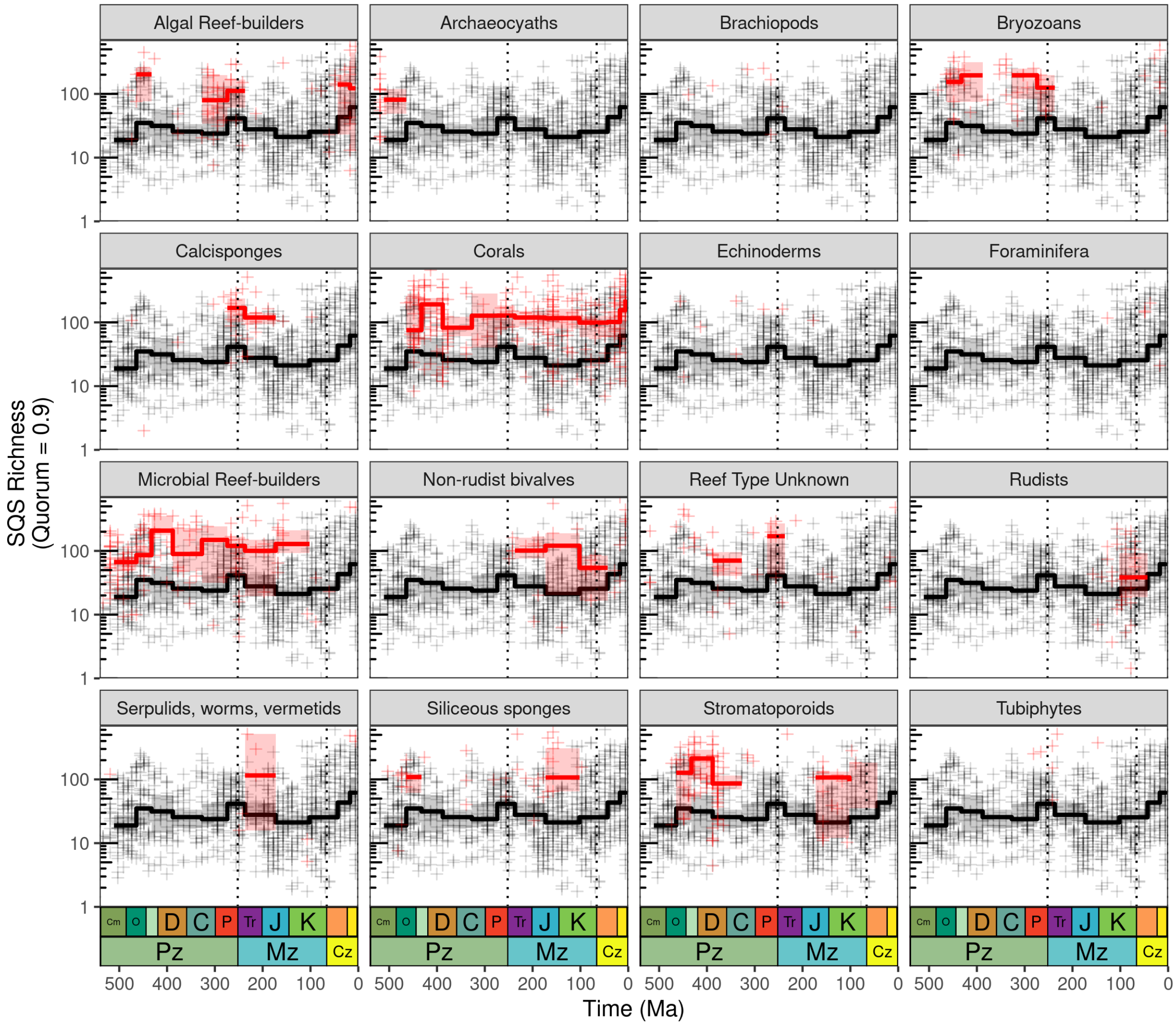

Fig. S19: Differences in diversity patterns (SQS, quorum = 0.9) for marine invertebrate animals between reef-supporting (red) and non-reef-supporting (black) regions (equal-area hexagonal/pentagonal grid cells with 1000 km spacings), showing patterns for additional kinds of reef-building organisms. Reef-supporting cells were identified as being associated with each particular kind of reef-building organism using the palaeocoordinates of reef sites listed in the PARED PaleoReefs database (*57*) (grid cells can appear in more than one panel if they contain more than one type of reef-building organism). Note logarithmic y-axes. Crosses represent SQS diversity estimates for individual grid cell regions, while trend lines represent medians and interquartile ranges of regional diversity for geological periods. Dashed lines represent boundaries between geological eras. PaleoDB collection data excludes those identified as unlithified and poorly-lithified-and-sieved deposits, but includes collections with no information on lithification style.

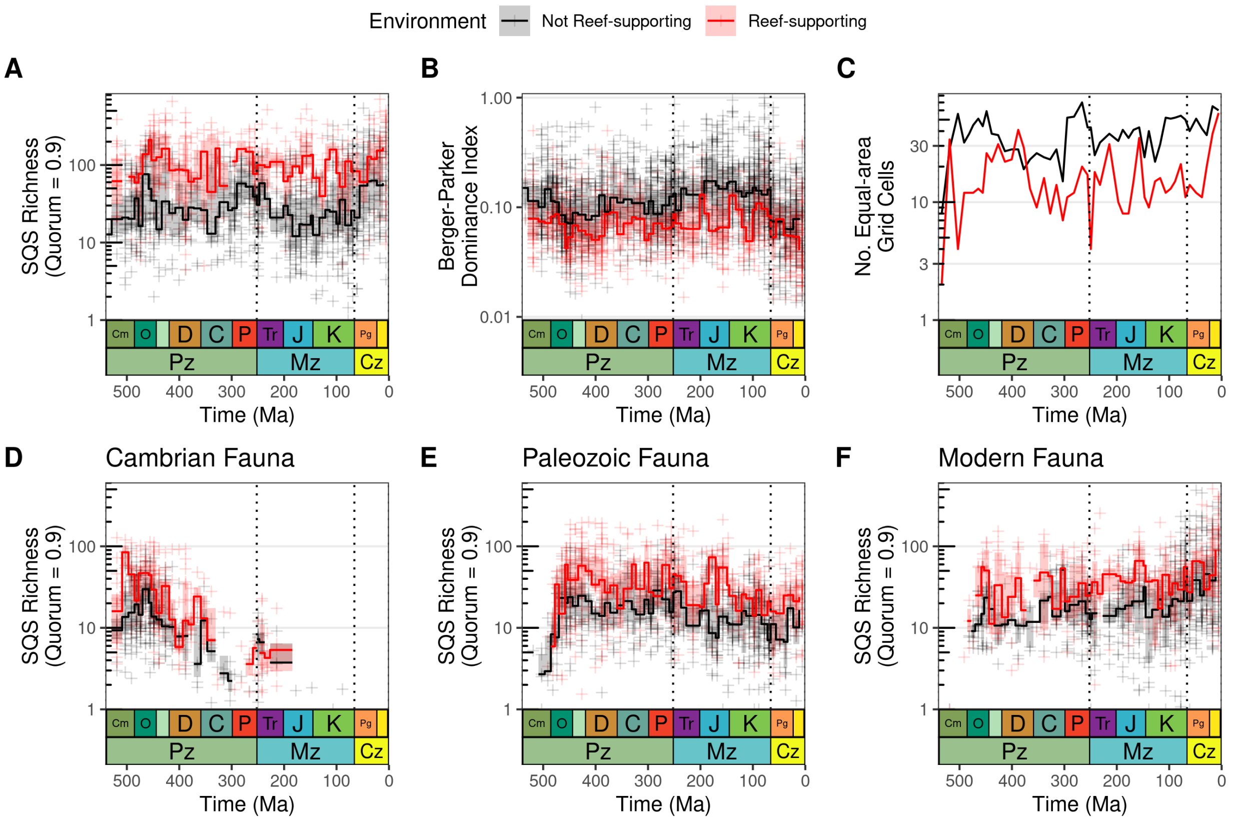

Fig. S20: As for main figure, but using equal-length time bins for summary trend-lines, rather than geological periods. Differences in Phanerozoic marine invertebrate animal diversity patterns between reef-supporting (red) and non-reef-supporting (black) regions (equal-area hexagonal/pentagonal grid cells with spacings of 1000 km for all panels in this figure), excluding collections explicitly identified as representing unlithified or poorly-lithified-and-sieved deposits, but retaining collections that have no information about lithification style. Dotted lines represent boundaries between geological eras. Note logarithmic y-axes. For panels A–B and D–F, crosses represent SQS diversity estimates for individual grid cell regions, while trend lines represent medians and interquartile ranges of regional diversity for equal-length bins. (A) Spatially-standardized Phanerozoic marine animal diversity, contrasting patterns for reef-supporting and non-reef-supporting regions. Note that in reef-supporting regions, levels of diversity have been broadly similar since the Ordovician, with no evidence for long-term, secular trends. In non-reef-supporting regions, by contrast, levels of diversity were similar from the Ordovician to the latest Cretaceous, when diversity rose fairly rapidly to a new, higher level that was sustained through the Cenozoic. However, this K/Pg increase is strongly associated with gastropods and unlithified sediments (see fig. S9). (B) Evenness, estimated using Berger-Parker dominance index (*49*), in reef-supporting and non-reef-supporting grid cells. (C) Counts of reef-supporting and non-reef-supporting cells through the Phanerozoic, using equal-length time bins. Panels (D–F) show patterns for Sepkoski's evolutionary faunas. (D) Cambrian Fauna (Trilobita, Linguliformea, Graptolithina, Conodonta); (E) Paleozoic Fauna (Anthozoa, Ostracoda, Rhynchonelliformea, Cephalopoda, Crinoidea); (F) Modern Fauna (Bryozoa, Bivalvia, Gastropoda, Echinoidea, Chondrichthyes).

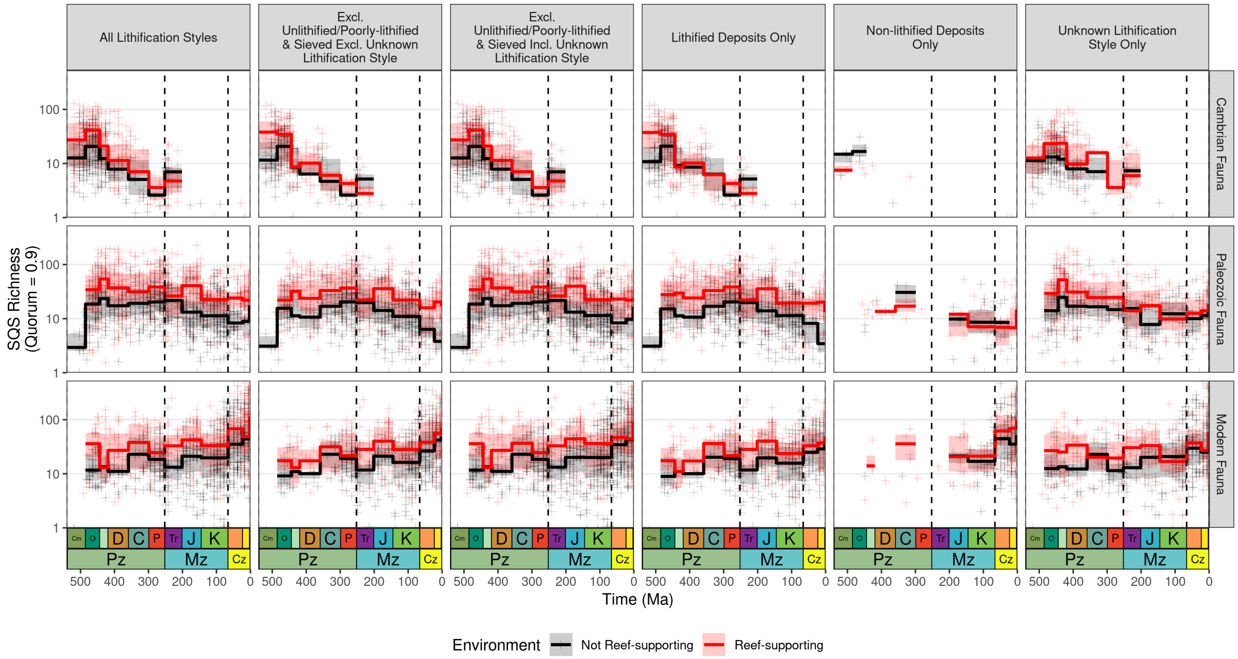

Fig. S21: SQS diversity for reef-supporting (red) and non-reef-supporting (black) regions (equal-area hexagonal/pentagonal grid cells with 1000 km spacings) within Sepkoski's three evolutionary faunas, partitioned by lithification style into PaleoDB collections representing lithified deposits, or those representing poorly-lithified or unlithified deposits.

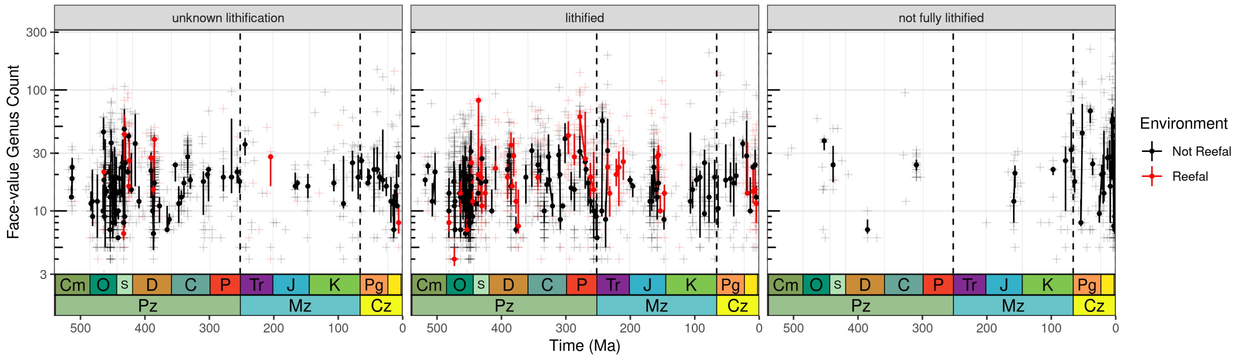

Fig. S22: Per-collection counts of genera for reefal and non-reefal facies, broken down by lithification style. Crosses represent counts of genera for individual collections, while trend-lines indicate medians and interquartile ranges for geological stages. See Supplementary Methods for details of quality criteria used to remove uninformative collections.

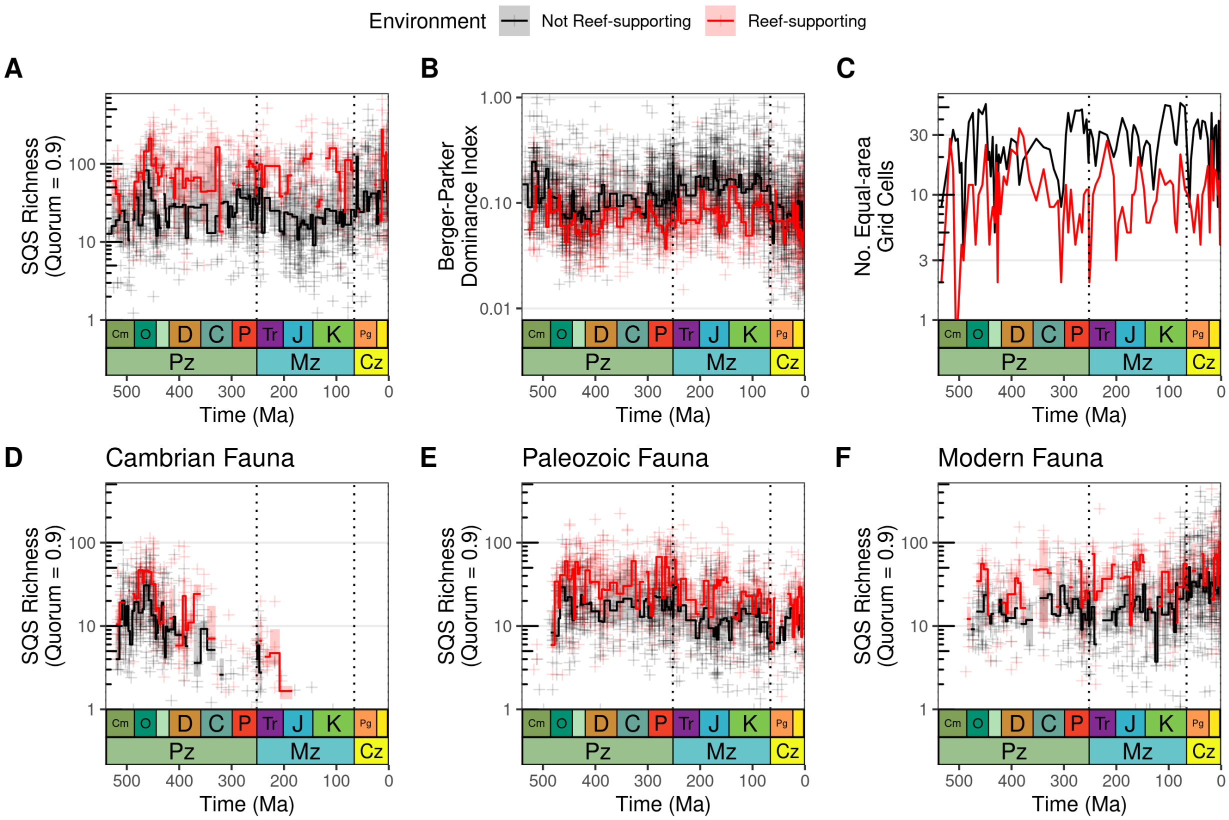

Fig. S23: As for main figure, but using stage-length time bins for analysis rather than equal-length time bins. Summary trend lines are at the scale of geological stages. Differences in Phanerozoic marine invertebrate animal diversity patterns between reef-supporting (red) and non-reef-supporting (black) regions (equal-area hexagonal/pentagonal grid cells with spacings of 1000 km for all panels in this figure), excluding collections explicitly identified as representing unlithified or poorly-lithified-and-sieved deposits, but retaining collections that have no information about lithification style. Dotted lines represent boundaries between geological eras. Note logarithmic y-axes. For panels A–B and D–F, crosses represent SQS diversity estimates for individual grid cell regions, while trend lines represent medians and interquartile ranges of regional diversity for stage-length bins. (A) Spatially-standardized Phanerozoic marine animal diversity, contrasting patterns for reef-supporting and non-reef-supporting regions. Note that in reef-supporting regions, levels of diversity have been broadly similar since the Ordovician, with no evidence for long-term, secular trends. In non-reef-supporting regions, by contrast, levels of diversity were similar from the Ordovician to the latest Cretaceous, when diversity rose fairly rapidly to a new, higher level that was sustained through the Cenozoic. However, this K/Pg increase is strongly associated with gastropods and unlithified sediments (see fig. S9). (B) Evenness, estimated using Berger-Parker dominance index (*49*), in reef-supporting and non-reef-supporting grid cells. (C) Counts of reef-supporting and non-reef-supporting cells through the Phanerozoic, using equal-length time bins. Panels (D–F) show patterns for Sepkoski's evolutionary faunas. (D) Cambrian Fauna (Trilobita, Linguliformea, Graptolithina, Conodonta); (E) Paleozoic Fauna (Anthozoa, Ostracoda, Rhynchonelliformea, Cephalopoda, Crinoidea); (F) Modern Fauna (Bryozoa, Bivalvia, Gastropoda, Echinoidea, Chondrichthyes).

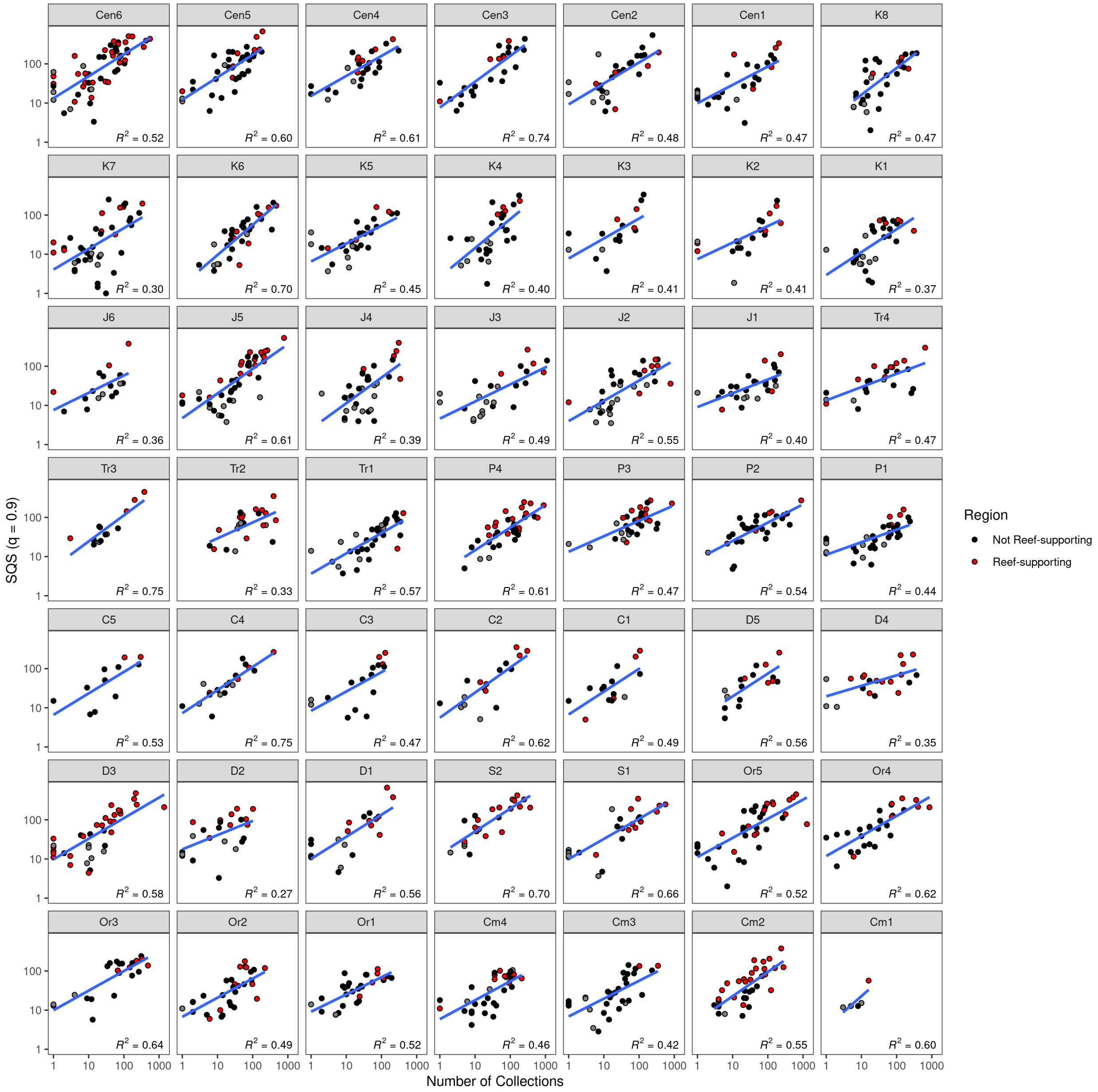

Fig. S24: Bivariate correlations between counts of collections per cell and SQS diversity (quorum 0.9) estimates for marine animals, for 1000 km equal-area hexagonal/pentagonal grid cells. Equal-length time bins are used, and data excludes unlithified and poorly-lithified and sieved deposits, but includes collections lacking metadata on lithification style.

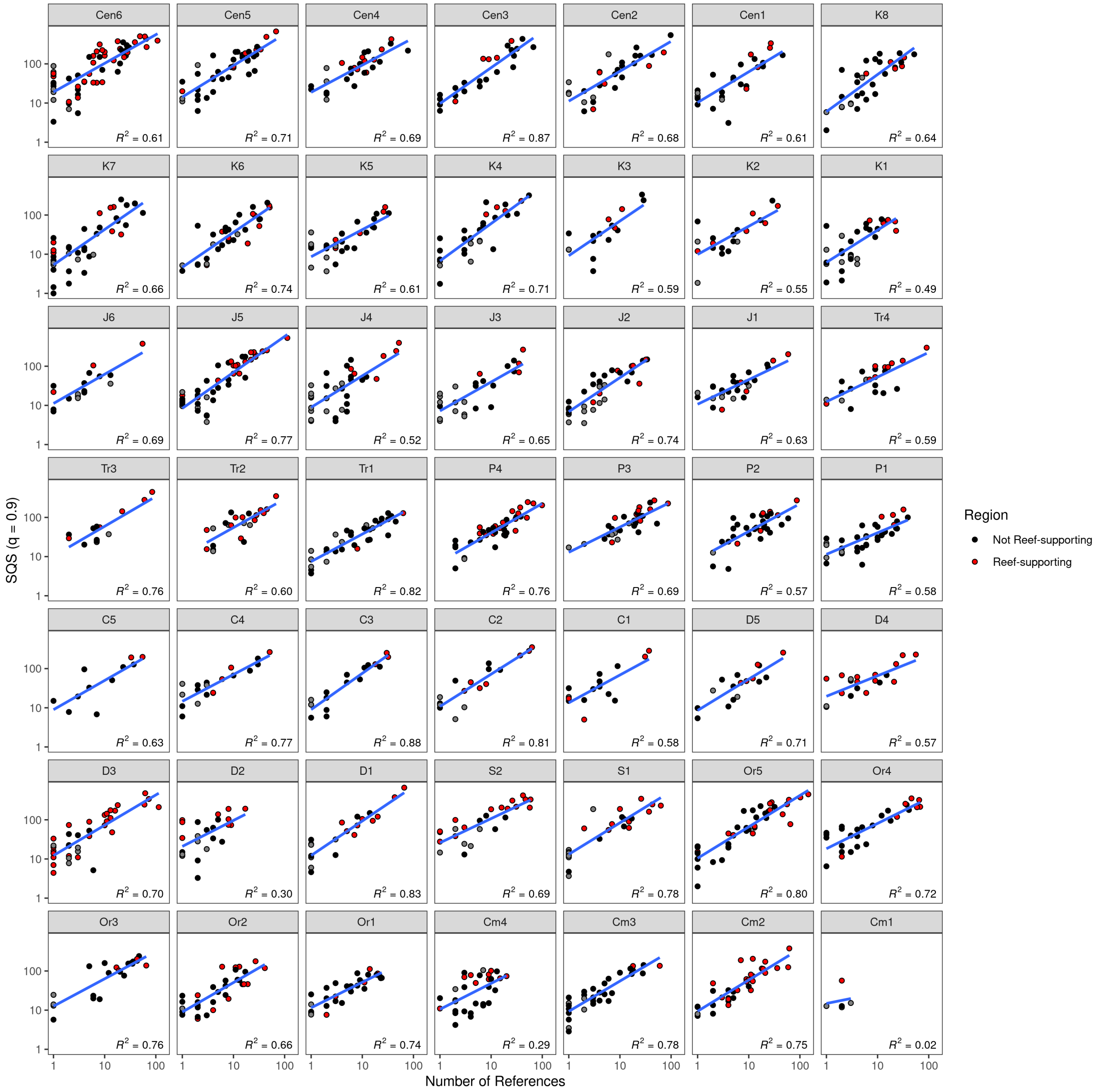

Fig. S25: Bivariate correlations between counts of references per cell and SQS diversity (quorum 0.9) estimates for marine animals, for 1000 km equal-area hexagonal/pentagonal grid cells. Equal-length time bins are used, and data excludes unlithified and poorly-lithified and sieved deposits, but includes collections lacking metadata on lithification style.

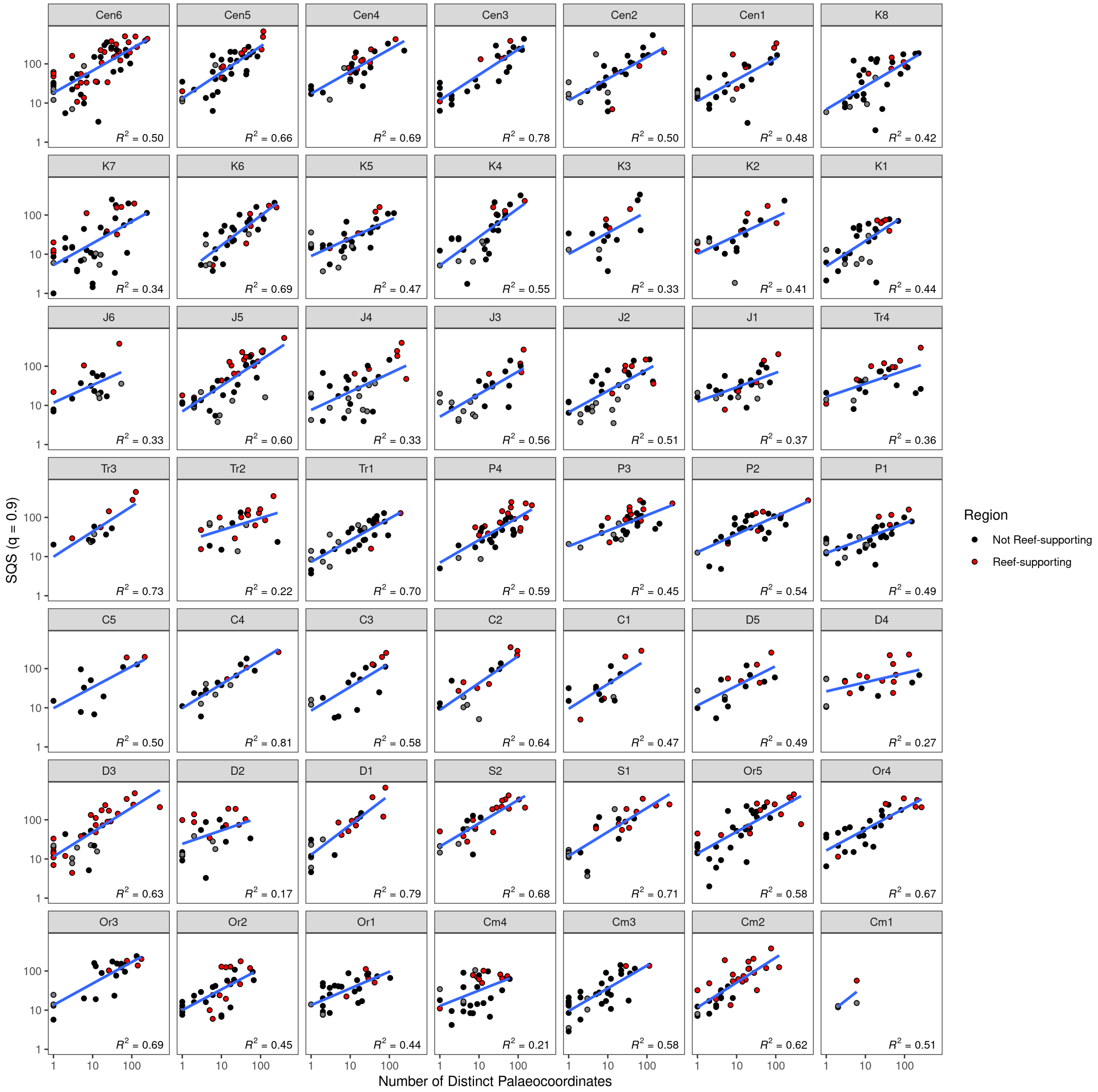

Fig. S26: Bivariate correlations between counts of distinct paleocoordinate locations per cell and SQS diversity (quorum 0.9) estimates for marine animals, for 1000 km equal-area hexagonal/pentagonal grid cells. Equal-length time bins are used, and data excludes unlithified and poorly-lithified and sieved deposits, but includes collections lacking metadata on lithification style.

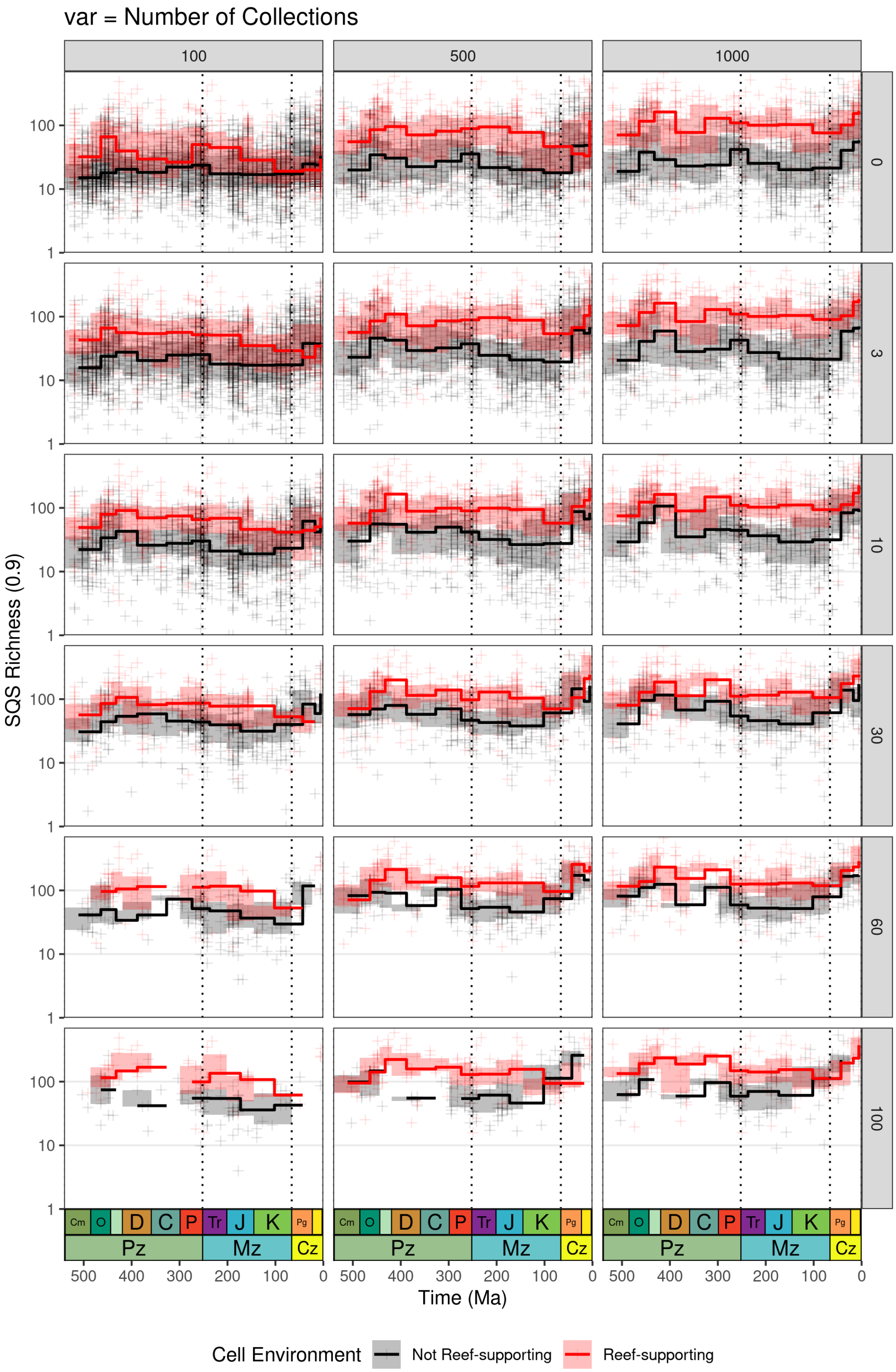

Fig. S27: The effect of imposing progressively higher filtering quotas of counts of collections per cell on patterns of reefs-supporting and non-reef-supporting diversity (quorum 0.9) estimates for marine animals, for 1000 km equal-area hexagonal/pentagonal grid cells. Equal-length time bins are used, and data excludes unlithified and poorly-lithified and sieved deposits, but includes collections lacking metadata on lithification style.

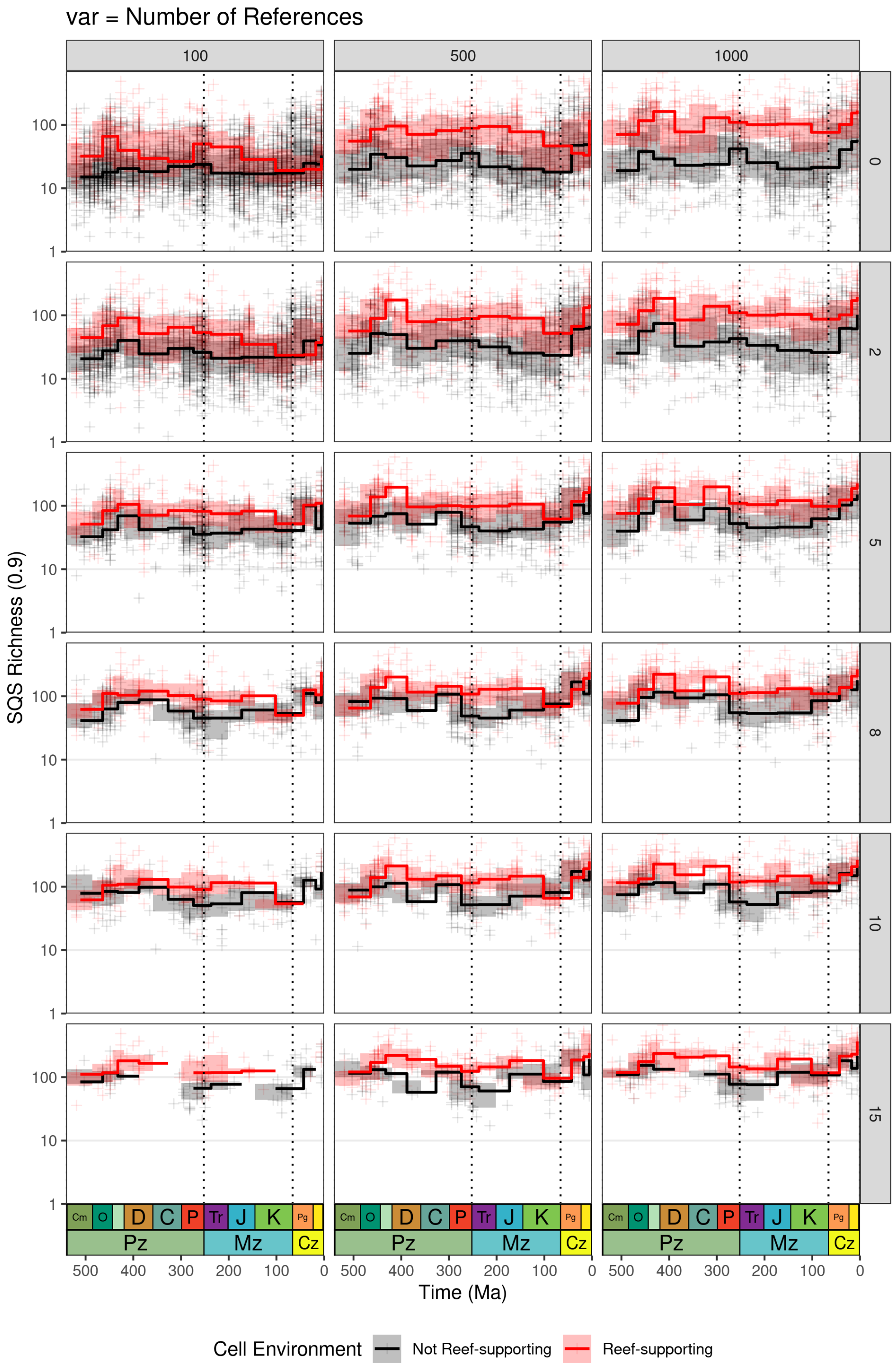

Fig. S28: The effect of imposing progressively higher filtering quotas of counts of references per cell on patterns of reefs-supporting and non-reef-supporting diversity (quorum 0.9) estimates for marine animals, for 1000 km equal-area hexagonal/pentagonal grid cells. Equal-length time bins are used, and data excludes unlithified and poorly-lithified and sieved deposits, but includes collections lacking metadata on lithification style.

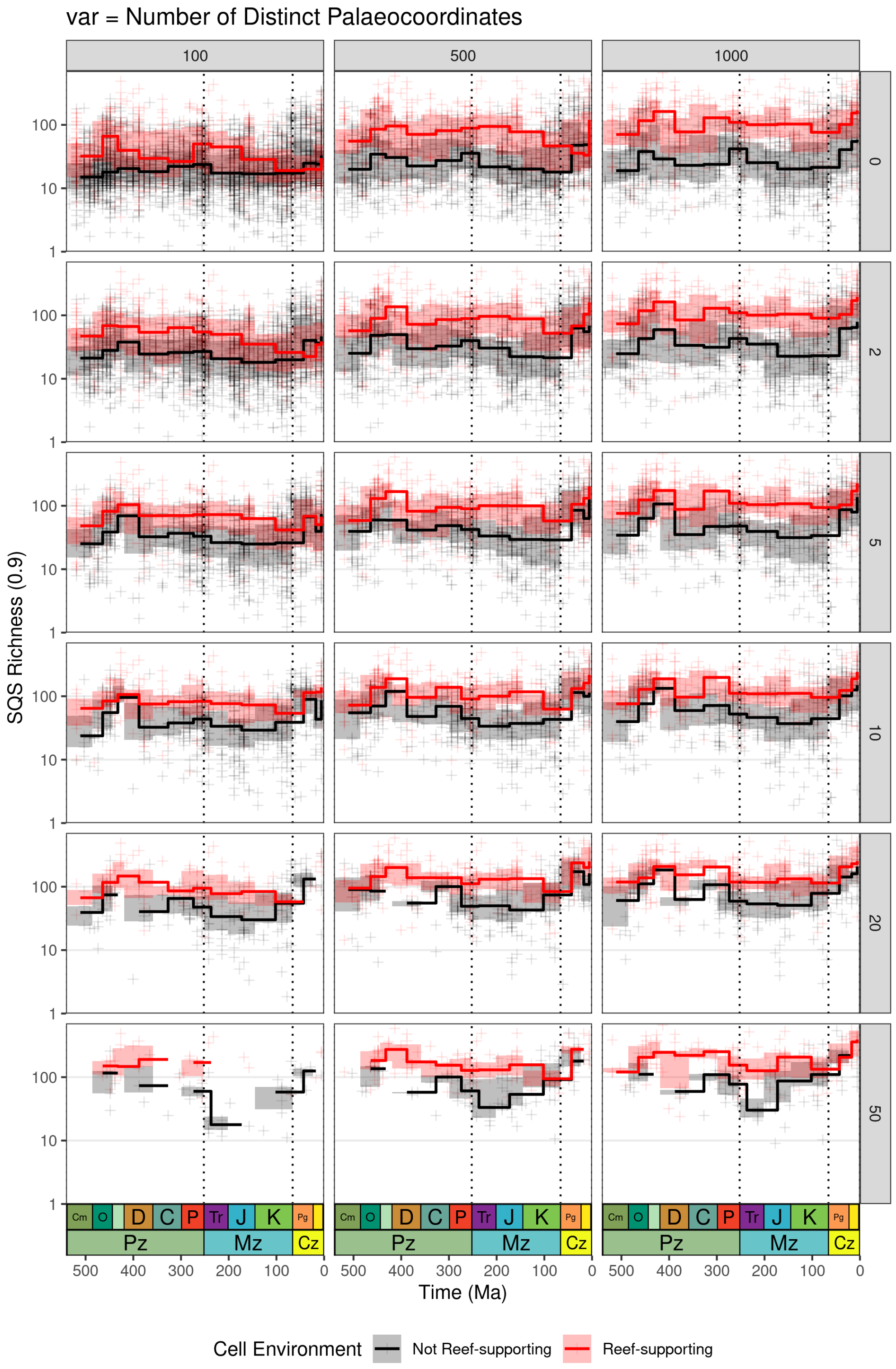

Fig. S29: The effect of imposing progressively higher filtering quotas of counts of distinct palaeocoordinate locations per cell on patterns of reefs-supporting and non-reef-supporting diversity (quorum 0.9) estimates for marine animals, for 1000 km equal-area hexagonal/pentagonal grid cells. Equal-length time bins are used, and data excludes unlithified and poorly-lithified and sieved deposits, but includes collections lacking metadata on lithification style.

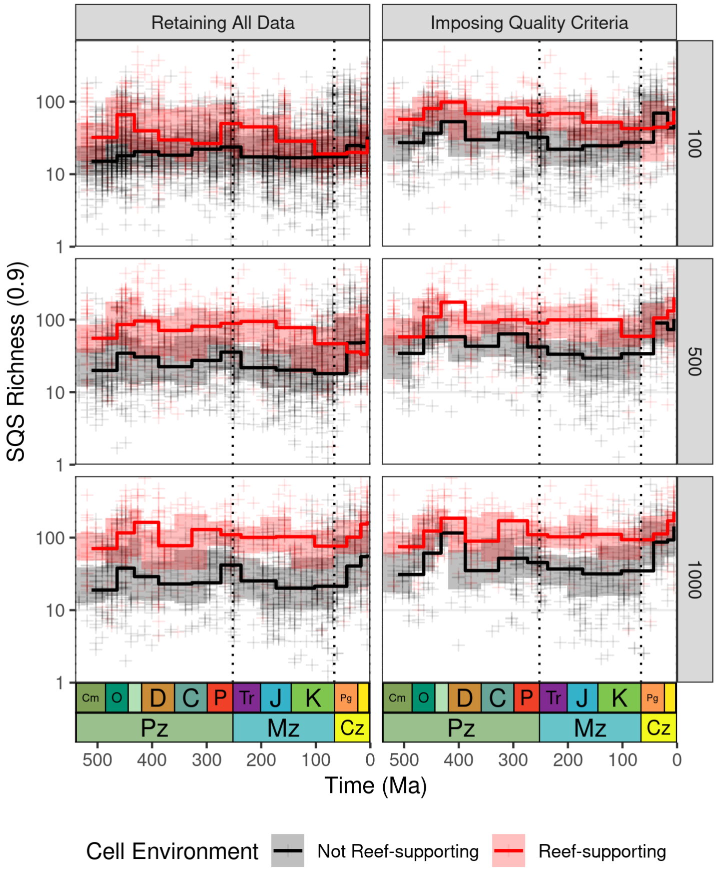

Fig. S30: The effect of imposing overall quality criteria filtering thresholds (at least 10 collections and 2 references) on patterns of reefs-supporting and non-reef-supporting diversity (quorum 0.9) estimates for marine animals, for 100 km to 2000 km equal-area hexagonal/pentagonal grid cells. Equal-length time bins are used, and data excludes unlithified and poorly-lithified and sieved deposits, but includes collections lacking metadata on lithification style.

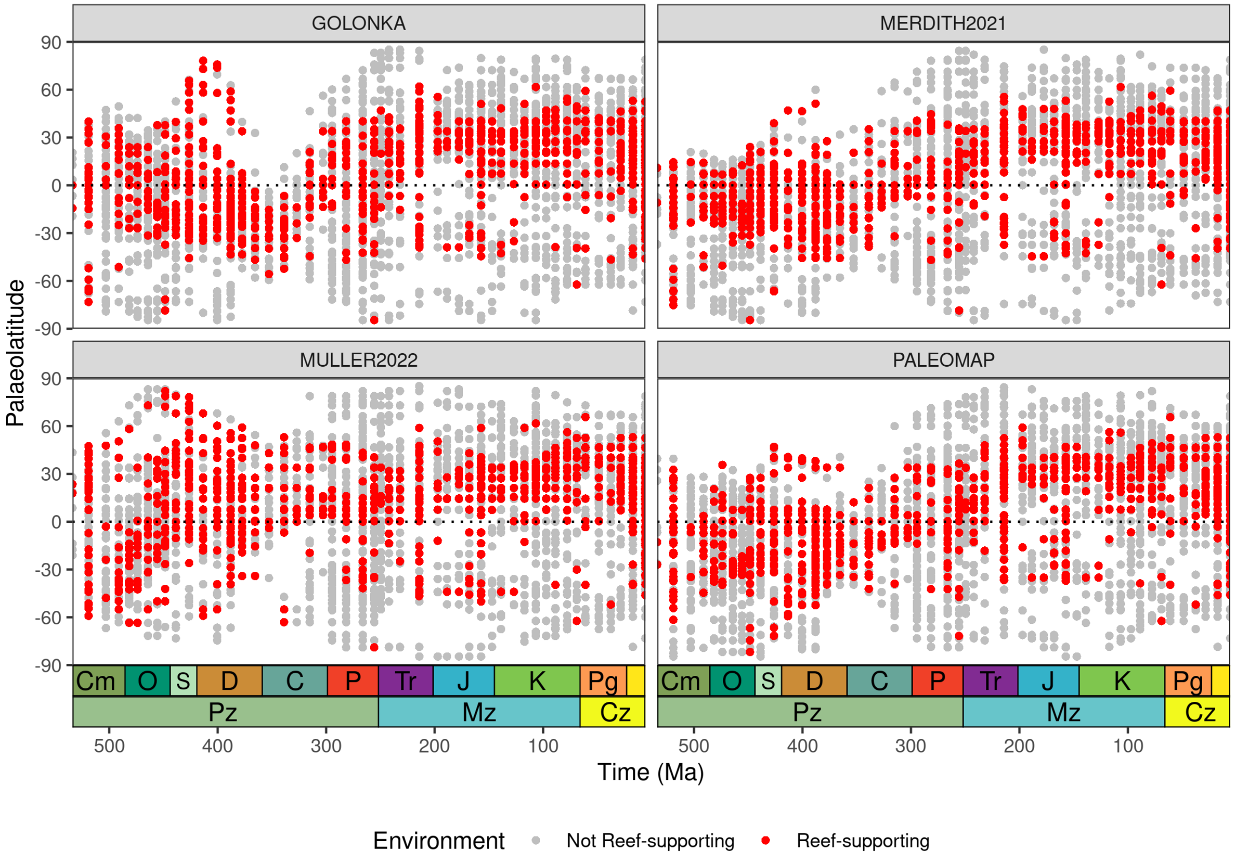

Fig. S31: Paleolatitudinal distribution of reef-supporting cells through the Phanerozoic for four different tectonic plate models (GOLONKA (*55*)); MERDITH2021 (*53*); MULLER2022 (*54*); PALEOMAP (*51*)).

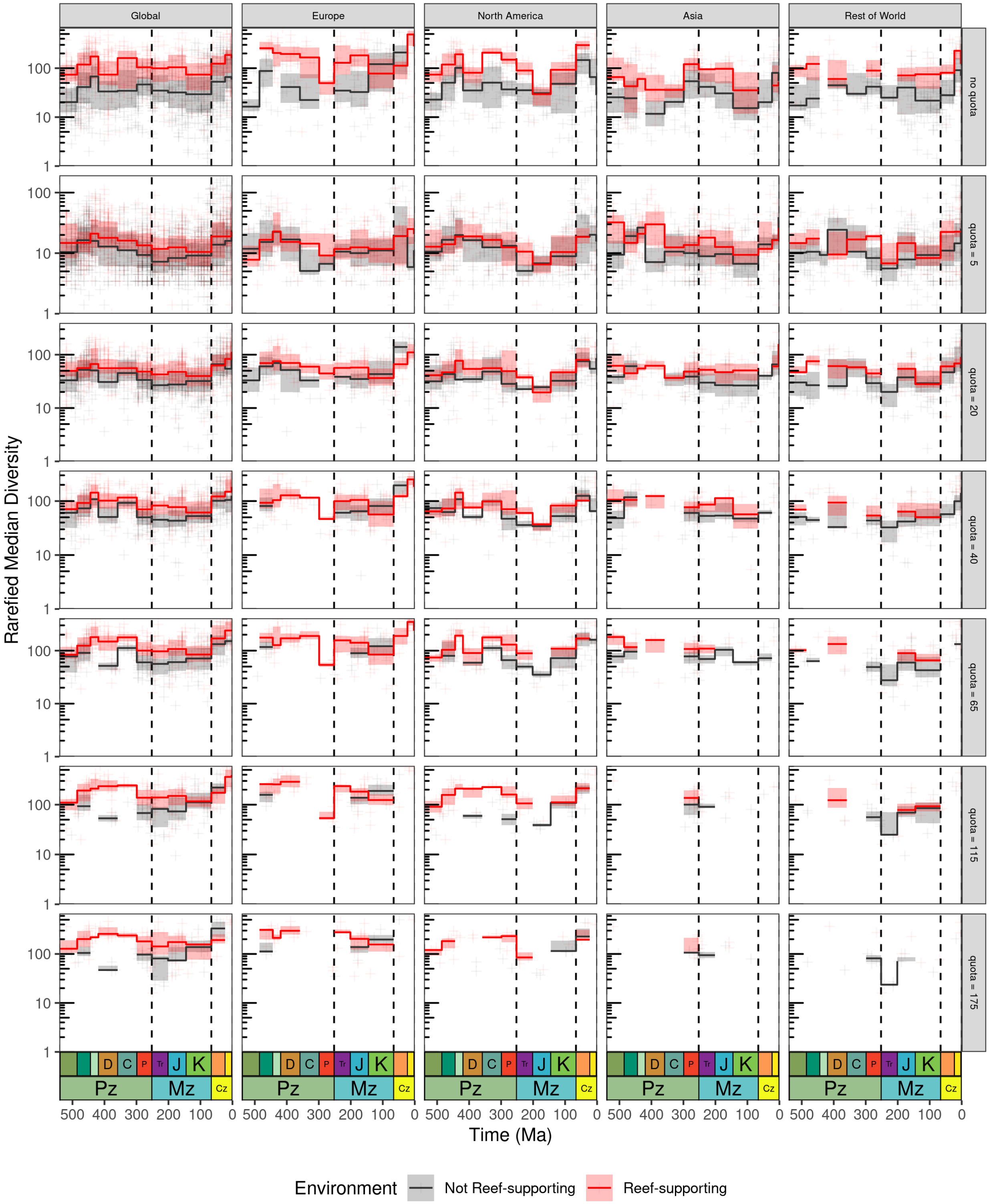

Fig. S32: Patterns of diversity for reef-supporting (red) and non-reef-supporting (black) regions (1000 km equal-area hexagonal grid cells) rarefied by PaleoDB fossil collections, with quotas from 5 to 100 collections. No quota is denoted using 'no quota'. Patterns are shown for global data and major modern continental regions (for region definitions see Table S6). Transparent crosses represent face-value counts of genera for grid cells, and lines with transparent ribbons represent medians and interquartile ranges for geological periods.

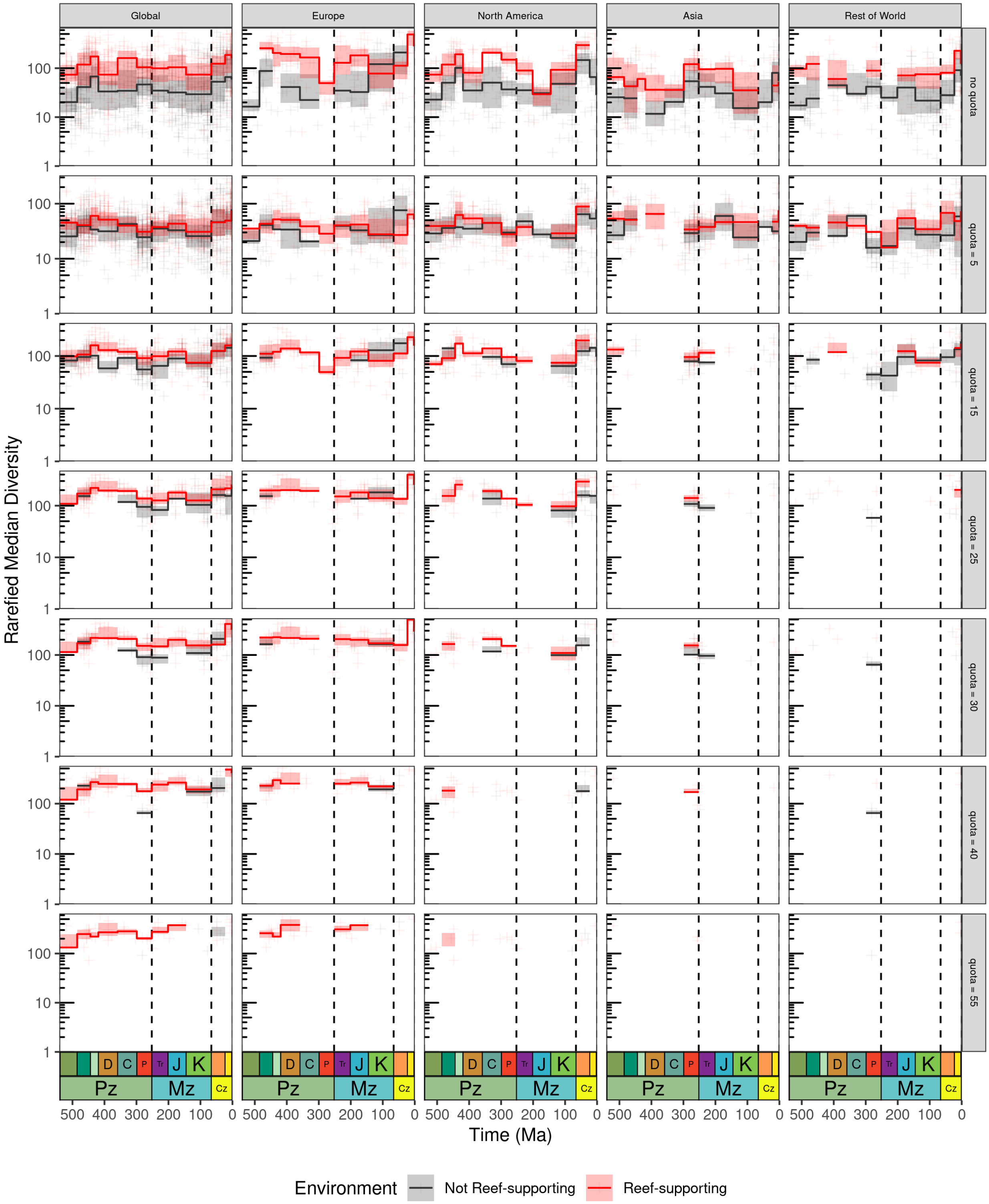

Fig. S33: Patterns of diversity for reef-supporting (red) and non-reef-supporting (black) regions (1000 km equal-area hexagonal grid cells) rarefied by reference (publications used to enter occurrences in the PaleoDB), with quotas from 5 to 50 references. No quota is denoted using 'no quota'. Patterns are shown for global data and major modern continental regions (for region definitions see Table S6). Transparent crosses represent face-value counts of genera for grid cells, and lines with transparent ribbons represent medians and interquartile ranges for geological periods.

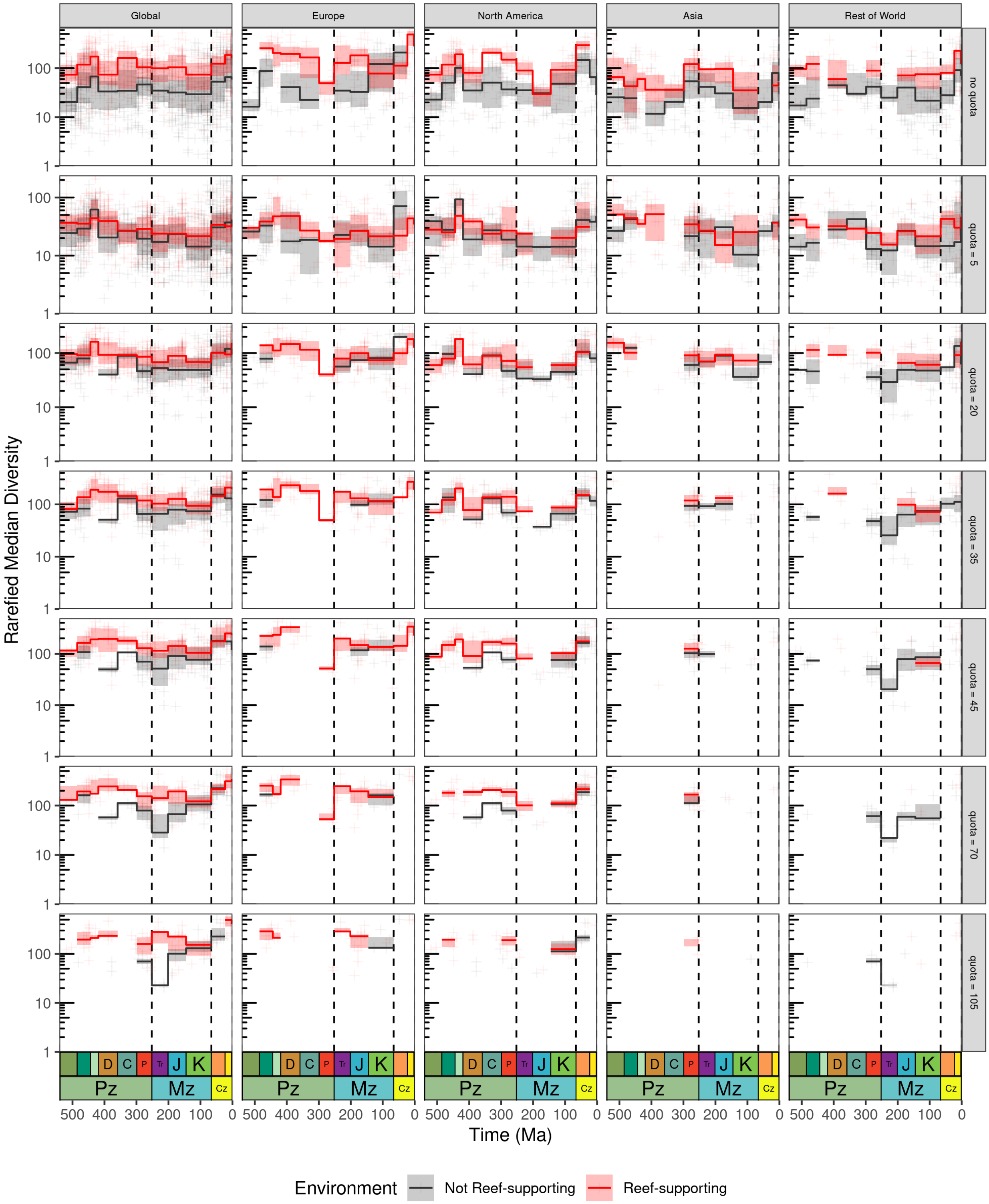

Fig. S34: Patterns of diversity for reef-supporting (red) and non-reef-supporting (black) regions (1000 km equal-area hexagonal grid cells) rarefied by unique paleocoordinate location represented by the sampled fossil localities, with quotas from 5 to 90 unique paleocoordinate locations. No quota is denoted using 'no quota'. Patterns are shown for global data and major modern continental regions (for region definitions see Table S6). Transparent crosses represent face-value counts of genera for grid cells, and lines with transparent ribbons represent medians and interquartile ranges for geological periods.

Table S1: ANOVA with Tukey HSD for collection-subsampled reefal/non-reefal counts of genera from reef-supporting/not cells

contrast	null.value	estimate	conf.low	conf.high	adj.p.value	significance
**100km cells, 10 collection quota**						
Non-reefal Facies in Reef-supporting Regions — Non-reefal Facies in Non-reef-supporting Regions	0	0.2198340	0.0912489	0.3484190	0.0001933	¡ 0.05
Reefal Facies in Reef-supporting Regions — Non-reefal Facies in Non-reef-supporting Regions	0	0.4365550	0.2510058	0.6221041	0.0000001	¡ 0.05
Reefal Facies in Reef-supporting Regions — Non-reefal Facies in Reef-supporting Regions	0	0.2167210	0.0147328	0.4187092	0.0320170	¡ 0.05
**100km cells, 20 collection quota**						
Non-reefal Facies in Reef-supporting Regions — Non-reefal Facies in Non-reef-supporting Regions	0	0.3806873	0.2398976	0.5214771	0.0000000	¡ 0.05
Reefal Facies in Reef-supporting Regions — Non-reefal Facies in Non-reef-supporting Regions	0	0.4679528	0.2489697	0.6869360	0.0000022	¡ 0.05
Reefal Facies in Reef-supporting Regions — Non-reefal Facies in Reef-supporting Regions	0	0.0872655	-0.1460348	0.3205658	0.6534511	n.s.
**100km cells, 40 collection quota**						
Non-reefal Facies in Reef-supporting Regions — Non-reefal Facies in Non-reef-supporting Regions	0	0.4329498	0.2437692	0.6221305	0.0000006	¡ 0.05
Reefal Facies in Reef-supporting Regions — Non-reefal Facies in Non-reef-supporting Regions	0	0.4283484	0.0901821	0.7665147	0.0087908	¡ 0.05
Reefal Facies in Reef-supporting Regions — Non-reefal Facies in Reef-supporting Regions	0	-0.0046014	-0.3559739	0.3467710	0.9994721	n.s.
**500km cells, 10 collection quota**						
Non-reefal Facies in Reef-supporting Regions — Non-reefal Facies in Non-reef-supporting Regions	0	0.1258884	0.0205169	0.2312600	0.0142277	¡ 0.05
Reefal Facies in Reef-supporting Regions — Non-reefal Facies in Non-reef-supporting Regions	0	0.3613306	0.2141535	0.5085077	0.0000000	¡ 0.05
Reefal Facies in Reef-supporting Regions — Non-reefal Facies in Reef-supporting Regions	0	0.2354422	0.0830366	0.3878478	0.0008836	¡ 0.05
**500km cells, 20 collection quota**						
Non-reefal Facies in Reef-supporting Regions — Non-reefal Facies in Non-reef-supporting Regions	0	0.1843311	0.0694723	0.2991898	0.0005213	¡ 0.05
Reefal Facies in Reef-supporting Regions — Non-reefal Facies in Non-reef-supporting Regions	0	0.4184998	0.2365887	0.6004108	0.0000003	¡ 0.05
Reefal Facies in Reef-supporting Regions — Non-reefal Facies in Reef-supporting Regions	0	0.2341687	0.0487741	0.4195633	0.0087380	¡ 0.05
**500km cells, 40 collection quota**						
Non-reefal Facies in Reef-supporting Regions — Non-reefal Facies in Non-reef-supporting Regions	0	0.3181156	0.1777590	0.4584722	0.0000005	¡ 0.05
Reefal Facies in Reef-supporting Regions — Non-reefal Facies in Non-reef-supporting Regions	0	0.4728717	0.1958378	0.7499055	0.0002124	¡ 0.05
Reefal Facies in Reef-supporting Regions — Non-reefal Facies in Reef-supporting Regions	0	0.1547561	-0.1222778	0.4317899	0.3878239	n.s.
**1000km cells, 10 collection quota**						
Non-reefal Facies in Reef-supporting Regions — Non-reefal Facies in Non-reef-supporting Regions	0	0.0933174	-0.0134937	0.2001285	0.1008646	n.s.
Reefal Facies in Reef-supporting Regions — Non-reefal Facies in Non-reef-supporting Regions	0	0.3275204	0.1813135	0.4737273	0.0000005	¡ 0.05
Reefal Facies in Reef-supporting Regions — Non-reefal Facies in Reef-supporting Regions	0	0.2342030	0.0862380	0.3821680	0.0006297	¡ 0.05
**1000km cells, 20 collection quota**						
Non-reefal Facies in Reef-supporting Regions — Non-reefal Facies in Non-reef-supporting Regions	0	0.1428853	0.0241935	0.2615770	0.0133822	¡ 0.05
Reefal Facies in Reef-supporting Regions — Non-reefal Facies in Non-reef-supporting Regions	0	0.3271998	0.1410713	0.5133283	0.0001218	¡ 0.05
Reefal Facies in Reef-supporting Regions — Non-reefal Facies in Reef-supporting Regions	0	0.1843145	-0.0014775	0.3701066	0.0523942	n.s.
**1000km cells, 40 collection quota**						
Non-reefal Facies in Reef-supporting Regions — Non-reefal Facies in Non-reef-supporting Regions	0	0.2674335	0.1283288	0.4065382	0.0000239	¡ 0.05
Reefal Facies in Reef-supporting Regions — Non-reefal Facies in Non-reef-supporting Regions	0	0.5016892	0.2510538	0.7523246	0.0000102	¡ 0.05
Reefal Facies in Reef-supporting Regions — Non-reefal Facies in Reef-supporting Regions	0	0.2342557	-0.0143949	0.4829063	0.0696616	n.s.

Table S2: Wilcoxon tests of whether there is a statistically significant difference in diversity among reef-supporting/non-reef-supporting regions, for the entire Phanerozoic, for several different sifting criteria (see Supplementary Methods) and equal-area hexagonal/pentagonal grid-cell sizes (spacings of 100 km, 500 km, 1000 km and 2000 km). Magnitude denotes strength of effect size.

grid cell size	group1	group2	n1	n2	statistic	p	p.signif	effect size (r)	magnitude	Median SQS (Not Reef-supporting)	Median SQS (Reef-supporting)	Richness Ratio
**All Lithification Styles**												
100km	Not Reef-supporting	Reef-supporting	4773	1200	367355.0	0.00e+00	****	0.2200	small	22.2	43.0	1.940
500km	Not Reef-supporting	Reef-supporting	2455	962	142011.5	0.00e+00	****	0.3590	moderate	30.4	77.8	2.560
1000km	Not Reef-supporting	Reef-supporting	1781	812	80017.0	0.00e+00	****	0.4480	moderate	32.7	106.0	3.240
**Excl. Unlithified/Poorly-lithified & Sieved Excl. Unknown Lithification Style**												
100km	Not Reef-supporting	Reef-supporting	3107	932	212882.5	0.00e+00	****	0.2250	small	19.3	32.4	1.680
500km	Not Reef-supporting	Reef-supporting	1768	821	95257.0	0.00e+00	****	0.3430	moderate	24.5	55.5	2.270
1000km	Not Reef-supporting	Reef-supporting	1316	714	58930.0	0.00e+00	****	0.3940	moderate	25.3	72.7	2.870
**Excl. Unlithified/Poorly-lithified & Sieved Incl. Unknown Lithification Style**												
100km	Not Reef-supporting	Reef-supporting	4659	1197	347298.5	0.00e+00	****	0.2290	small	21.6	42.3	1.960
500km	Not Reef-supporting	Reef-supporting	2432	960	137973.5	0.00e+00	****	0.3560	moderate	30.2	76.3	2.530
1000km	Not Reef-supporting	Reef-supporting	1770	811	77527.0	0.00e+00	****	0.4440	moderate	31.7	103.0	3.250
**Lithified Deposits Only**												
100km	Not Reef-supporting	Reef-supporting	2834	908	197836.0	0.00e+00	****	0.2300	small	18.3	28.1	1.540
500km	Not Reef-supporting	Reef-supporting	1640	804	91015.0	0.00e+00	****	0.3320	moderate	23.1	48.9	2.120
1000km	Not Reef-supporting	Reef-supporting	1242	703	52912.0	0.00e+00	****	0.3970	moderate	24.2	68.9	2.850
**Non-lithified Deposits Only**												
100km	Not Reef-supporting	Reef-supporting	599	113	9831.5	7.18e-01	ns	0.0177	small	30.4	29.4	0.967
500km	Not Reef-supporting	Reef-supporting	334	152	7142.5	3.54e-01	ns	0.0568	small	30.7	37.9	1.230
1000km	Not Reef-supporting	Reef-supporting	236	162	5566.5	2.72e-01	ns	0.0734	small	32.8	39.0	1.190
**Unknown Lithification Style Only**												
100km	Not Reef-supporting	Reef-supporting	2153	601	103280.5	5.80e-06	****	0.1300	small	18.4	26.4	1.430
500km	Not Reef-supporting	Reef-supporting	1273	638	49621.5	0.00e+00	****	0.2160	small	22.1	37.8	1.710
1000km	Not Reef-supporting	Reef-supporting	970	594	32007.0	0.00e+00	****	0.2820	small	23.2	45.4	1.960

Table S3: Wilcoxon tests of whether there is a statistically significant difference in diversity among reef-supporting/non-reef-supporting regions, within pre- and post-K/Pg intervals, for several different sifting criteria (see Supplementary Methods) and equal-area hexagonal/pentagonal grid-cell sizes (spacings of 100 km, 500 km, 1000 km and 2000 km). Magnitude denotes strength of effect size.

grid cell size	pre-/post-K/Pg	group1	group2	n1	n2	statistic	p	p.signif	effect size (r)	magnitude	Median SQS (Not Reef-supporting)	Median SQS (Reef-supporting)	Richness Ratio
**All Lithification Styles**													
100km	Pre-K/Pg	Not Reef-supporting	Reef-supporting	3863	961	221030.5	0.00e+00	****	2.71e-01	small	20.4	44.9	2.200
100km	Post-K/Pg	Not Reef-supporting	Reef-supporting	910	239	16929.0	9.21e-01	ns	4.59e-03	small	38.0	33.5	0.882
500km	Pre-K/Pg	Not Reef-supporting	Reef-supporting	2035	787	90550.5	0.00e+00	****	4.07e-01	moderate	26.5	74.9	2.830
500km	Post-K/Pg	Not Reef-supporting	Reef-supporting	420	175	4609.0	3.71e-03	**	1.84e-01	small	54.8	127.0	2.320
1000km	Post-K/Pg	Not Reef-supporting	Reef-supporting	278	148	2657.0	1.70e-06	****	3.32e-01	moderate	62.4	161.0	2.580
1000km	Pre-K/Pg	Not Reef-supporting	Reef-supporting	1503	664	51307.5	0.00e+00	****	4.82e-01	moderate	29.4	101.0	3.440
**Excl. Unlithified/Poorly-lithified & Sieved Excl. Unknown Lithification Style**													
100km	Pre-K/Pg	Not Reef-supporting	Reef-supporting	2615	771	136727.5	0.00e+00	****	2.64e-01	small	18.0	34.7	1.930
100km	Post-K/Pg	Not Reef-supporting	Reef-supporting	492	161	7968.5	9.99e-01	ns	9.13e-05	small	30.2	23.5	0.778
500km	Pre-K/Pg	Not Reef-supporting	Reef-supporting	1484	679	63334.5	0.00e+00	****	3.88e-01	moderate	22.4	55.7	2.490
500km	Post-K/Pg	Not Reef-supporting	Reef-supporting	284	142	2973.0	3.33e-01	ns	7.35e-02	small	41.9	43.4	1.040
1000km	Post-K/Pg	Not Reef-supporting	Reef-supporting	198	125	1947.0	6.75e-02	ns	1.53e-01	small	47.3	72.0	1.520
1000km	Pre-K/Pg	Not Reef-supporting	Reef-supporting	1118	589	38529.0	0.00e+00	****	4.36e-01	moderate	24.0	73.4	3.060
**Excl. Unlithified/Poorly-lithified & Sieved Incl. Unknown Lithification Style**													
100km	Pre-K/Pg	Not Reef-supporting	Reef-supporting	3853	961	220222.5	0.00e+00	****	2.71e-01	small	20.3	45.0	2.220
100km	Post-K/Pg	Not Reef-supporting	Reef-supporting	806	236	13542.5	7.05e-01	ns	1.90e-02	small	34.7	29.5	0.850
500km	Pre-K/Pg	Not Reef-supporting	Reef-supporting	2033	786	90166.5	0.00e+00	****	4.08e-01	moderate	26.5	75.2	2.840
500km	Post-K/Pg	Not Reef-supporting	Reef-supporting	399	174	4204.0	1.53e-01	ns	9.60e-02	small	54.6	93.9	1.720
1000km	Post-K/Pg	Not Reef-supporting	Reef-supporting	267	147	2301.0	2.14e-04	***	2.76e-01	small	61.9	141.0	2.280
1000km	Pre-K/Pg	Not Reef-supporting	Reef-supporting	1503	664	51348.5	0.00e+00	****	4.80e-01	moderate	29.2	98.1	3.360
**Lithified Deposits Only**													
100km	Pre-K/Pg	Not Reef-supporting	Reef-supporting	2528	763	136156.5	0.00e+00	****	2.56e-01	small	18.0	32.5	1.810
100km	Post-K/Pg	Not Reef-supporting	Reef-supporting	306	145	5080.0	7.49e-01	ns	2.18e-02	small	25.5	22.9	0.898
500km	Pre-K/Pg	Not Reef-supporting	Reef-supporting	1438	675	64087.0	0.00e+00	****	3.76e-01	moderate	22.2	54.8	2.470
500km	Post-K/Pg	Not Reef-supporting	Reef-supporting	202	129	1994.5	4.78e-01	ns	6.42e-02	small	38.1	27.2	0.714
1000km	Pre-K/Pg	Not Reef-supporting	Reef-supporting	1096	587	37835.5	0.00e+00	****	4.28e-01	moderate	23.8	70.7	2.970
1000km	Post-K/Pg	Not Reef-supporting	Reef-supporting	146	116	1028.0	1.69e-01	ns	1.39e-01	small	36.0	41.6	1.160
**Non-lithified Deposits Only**													
100km	Post-K/Pg	Not Reef-supporting	Reef-supporting	409	64	2756.0	4.25e-02	*	1.22e-01	small	36.7	61.0	1.660
100km	Pre-K/Pg	Not Reef-supporting	Reef-supporting	190	49	1591.5	8.41e-01	ns	1.70e-02	small	17.9	18.0	1.010
500km	Pre-K/Pg	Not Reef-supporting	Reef-supporting	134	72	1657.5	6.67e-01	ns	3.94e-02	small	18.0	19.8	1.100
500km	Post-K/Pg	Not Reef-supporting	Reef-supporting	200	80	1391.5	4.96e-03	**	2.33e-01	small	44.0	104.0	2.360
1000km	Post-K/Pg	Not Reef-supporting	Reef-supporting	144	77	1125.5	2.85e-03	**	2.72e-01	small	49.1	102.0	2.080
1000km	Pre-K/Pg	Not Reef-supporting	Reef-supporting	92	85	1300.5	8.74e-01	ns	1.59e-02	small	18.0	21.7	1.210
**Unknown Lithification Style Only**													
100km	Pre-K/Pg	Not Reef-supporting	Reef-supporting	1683	459	58663.5	0.00e+00	****	1.75e-01	small	16.9	28.0	1.660
100km	Post-K/Pg	Not Reef-supporting	Reef-supporting	470	142	6221.0	1.57e-01	ns	9.22e-02	small	30.0	22.0	0.733
500km	Pre-K/Pg	Not Reef-supporting	Reef-supporting	1028	499	32407.0	0.00e+00	****	2.61e-01	small	19.8	37.4	1.890
500km	Post-K/Pg	Not Reef-supporting	Reef-supporting	245	139	1728.0	9.52e-01	ns	5.53e-03	small	40.2	38.4	0.955
1000km	Pre-K/Pg	Not Reef-supporting	Reef-supporting	789	467	19968.5	0.00e+00	****	3.35e-01	moderate	20.2	46.0	2.280
1000km	Post-K/Pg	Not Reef-supporting	Reef-supporting	181	127	1337.5	7.71e-01	ns	2.80e-02	small	55.5	39.0	0.703

Table S4: Wilcoxon tests of whether there is a statistically significant difference in diversity between the pre- and post-K/Pg intervals within reef-supporting/non-reef-supporting regions, for several different sifting criteria (see Supplementary Methods) and equal-area hexagonal/pentagonal grid-cell sizes (spacings of 100 km, 500 km, 1000 km and 2000 km). Magnitude denotes strength of effect size.

grid cell size	environment	group1	group2	n1	n2	statistic	p	p.signif	effect size (r)	magnitude	Median SQS (Pre-K/Pg)	Median SQS (Post-K/Pg)	Richness Ratio
**All Lithification Styles**													
100km	Not Reef-supporting	Post-K/Pg	Pre-K/Pg	910	3863	489599.5	0.00e+00	****	0.22600	small	20.4	38.0	1.860
100km	Reef-supporting	Post-K/Pg	Pre-K/Pg	239	961	16558.5	5.40e-01	ns	0.02770	small	44.9	33.5	0.746
500km	Not Reef-supporting	Post-K/Pg	Pre-K/Pg	420	2035	127528.5	0.00e+00	****	0.25000	small	26.5	54.8	2.070
500km	Reef-supporting	Post-K/Pg	Pre-K/Pg	175	787	14777.0	3.11e-02	*	0.10200	small	74.9	127.0	1.700
1000km	Not Reef-supporting	Post-K/Pg	Pre-K/Pg	278	1503	73372.5	0.00e+00	****	0.22000	small	29.4	62.4	2.120
1000km	Reef-supporting	Post-K/Pg	Pre-K/Pg	148	664	13683.5	6.54e-04	***	0.16900	small	101.0	161.0	1.590
**Excl. Unlithified/Poorly-lithified & Sieved Excl. Unknown Lithification Style**													
100km	Not Reef-supporting	Post-K/Pg	Pre-K/Pg	492	2615	198568.0	0.00e+00	****	0.16100	small	18.0	30.2	1.680
100km	Reef-supporting	Post-K/Pg	Pre-K/Pg	161	771	9940.0	1.21e-01	ns	0.07800	small	34.7	23.5	0.677
500km	Not Reef-supporting	Post-K/Pg	Pre-K/Pg	284	1484	60912.0	0.00e+00	****	0.19000	small	22.4	41.9	1.870
500km	Reef-supporting	Post-K/Pg	Pre-K/Pg	142	679	8163.5	3.55e-01	ns	0.04770	small	55.7	43.4	0.779
1000km	Not Reef-supporting	Post-K/Pg	Pre-K/Pg	198	1118	34334.0	2.80e-06	****	0.18000	small	24.0	47.3	1.970
1000km	Reef-supporting	Post-K/Pg	Pre-K/Pg	125	589	7489.0	9.23e-01	ns	0.00529	small	73.4	72.0	0.981
**Excl. Unlithified/Poorly-lithified & Sieved Incl. Unknown Lithification Style**													
100km	Not Reef-supporting	Post-K/Pg	Pre-K/Pg	806	3853	376412.5	0.00e+00	****	0.17500	small	20.3	34.7	1.710
100km	Reef-supporting	Post-K/Pg	Pre-K/Pg	236	961	14558.5	5.06e-02	ns	0.08880	small	45.0	29.5	0.656
500km	Not Reef-supporting	Post-K/Pg	Pre-K/Pg	399	2033	113032.0	0.00e+00	****	0.24000	small	26.5	54.6	2.060
500km	Reef-supporting	Post-K/Pg	Pre-K/Pg	174	786	11738.0	6.31e-01	ns	0.02290	small	75.2	93.9	1.250
1000km	Not Reef-supporting	Post-K/Pg	Pre-K/Pg	267	1503	62011.5	0.00e+00	****	0.20400	small	29.2	61.9	2.120
1000km	Reef-supporting	Post-K/Pg	Pre-K/Pg	147	664	11375.5	3.56e-02	*	0.10600	small	98.1	141.0	1.440
**Lithified Deposits Only**													
100km	Not Reef-supporting	Post-K/Pg	Pre-K/Pg	306	2528	114557.0	1.71e-04	***	0.09790	small	18.0	25.5	1.420
100km	Reef-supporting	Post-K/Pg	Pre-K/Pg	145	763	9740.0	2.39e-02	*	0.11300	small	32.5	22.9	0.705
500km	Not Reef-supporting	Post-K/Pg	Pre-K/Pg	202	1438	33073.0	4.92e-04	***	0.12100	small	22.2	38.1	1.720
500km	Reef-supporting	Post-K/Pg	Pre-K/Pg	129	675	5668.0	8.08e-05	****	0.20300	small	54.8	27.2	0.496
1000km	Not Reef-supporting	Post-K/Pg	Pre-K/Pg	146	1096	16684.5	2.36e-02	*	0.09100	small	23.8	36.0	1.510
1000km	Reef-supporting	Post-K/Pg	Pre-K/Pg	116	587	5935.5	8.69e-02	ns	0.09430	small	70.7	41.6	0.588
**Non-lithified Deposits Only**													
100km	Not Reef-supporting	Post-K/Pg	Pre-K/Pg	409	190	19686.0	0.00e+00	****	0.31100	moderate	17.9	36.7	2.050
100km	Reef-supporting	Post-K/Pg	Pre-K/Pg	64	49	628.0	1.09e-04	***	0.51800	large	18.0	61.0	3.390
500km	Not Reef-supporting	Post-K/Pg	Pre-K/Pg	200	134	5800.0	5.00e-07	****	0.37100	moderate	18.0	44.0	2.440
500km	Reef-supporting	Post-K/Pg	Pre-K/Pg	80	72	1422.0	7.00e-07	****	0.54300	large	19.8	104.0	5.250
1000km	Not Reef-supporting	Post-K/Pg	Pre-K/Pg	144	92	2945.5	3.83e-05	****	0.36000	moderate	18.0	49.1	2.730
1000km	Reef-supporting	Post-K/Pg	Pre-K/Pg	77	85	1806.5	0.00e+00	****	0.58500	large	21.7	102.0	4.700
**Unknown Lithification Style Only**													
100km	Not Reef-supporting	Post-K/Pg	Pre-K/Pg	470	1683	85961.5	0.00e+00	****	0.19400	small	16.9	30.0	1.780
100km	Reef-supporting	Post-K/Pg	Pre-K/Pg	142	459	6058.5	3.80e-01	ns	0.05390	small	28.0	22.0	0.786
500km	Not Reef-supporting	Post-K/Pg	Pre-K/Pg	245	1028	25696.0	2.00e-07	****	0.22600	small	19.8	40.2	2.030
500km	Reef-supporting	Post-K/Pg	Pre-K/Pg	139	499	4494.5	7.57e-01	ns	0.01930	small	37.4	38.4	1.030
1000km	Not Reef-supporting	Post-K/Pg	Pre-K/Pg	181	789	17307.0	1.00e-07	****	0.26300	small	20.2	55.5	2.750
1000km	Reef-supporting	Post-K/Pg	Pre-K/Pg	127	467	4000.5	5.36e-01	ns	0.04070	small	46.0	39.0	0.848

Table S5: Effects of gastropods on K/Pg diversity increase (testing whether diversity is greater in Cz than it is in Pz-Mz)

environment	group1	group2	n1	n2	statistic	p	p.signif	effect size (r)	magnitude	Median SQS (Post-K/Pg)	Median SQS (Pre-K/Pg)	Richness Ratio
**Gastropods**												
Not Reef-supporting	Pre-K/Pg	Post-K/Pg	144	94	2776.0	0.00e+00	****	0.4980	moderate	35.8	11.5	3.11
Reef-supporting	Pre-K/Pg	Post-K/Pg	121	46	1166.5	0.00e+00	****	0.4480	moderate	57.0	21.7	2.63
**Marine Invertebrates**												
Not Reef-supporting	Pre-K/Pg	Post-K/Pg	986	172	63097.0	1.00e-07	****	0.1580	small	34.7	20.3	1.71
Reef-supporting	Pre-K/Pg	Post-K/Pg	516	119	28869.5	3.10e-01	ns	0.0403	small	55.5	47.7	1.16
**Marine Invertebrates Excluding Gastropods**												
Not Reef-supporting	Pre-K/Pg	Post-K/Pg	967	151	62501.5	4.41e-03	**	0.0852	small	26.4	20.1	1.31
Reef-supporting	Pre-K/Pg	Post-K/Pg	511	113	29604.0	6.73e-01	ns	0.0169	small	49.3	45.8	1.08

Table S6: Definitions of modern geographic regions

**Modern Geographic Region**	**Countries Included**
North America (NAm)	United States, Canada, Mexico
Asia (AS)	China, Hong Kong SAR China, Mongolia, South Korea, Russia, North Korea, Japan, Taiwan, Democratic People's Republic of Korea, Afghanistan, Armenia, Azerbaijan, Kazakhstan, Bahrain, Bhutan, Cambodia, Laos, India, Bangladesh, Indonesia, Iran, Iraq, Israel, Kuwait, Qatar, Malaysia, Pakistan, Philippines, Taiwan, Province of China, Syria, Yemen, Uzbekistan, Vietnam, Thailand, Tajikistan, Republic of Korea, Myanmar (Burma), Kyrgyzstan, Jordan, Lao People's Democratic Republic, Lebanon, Palestinian Territories, Turkey, Nepal, Turkmenistan, Saudi Arabia, Oman, United Arab Emirates, Sri Lanka
Europe (EU)	United Kingdom, France, Germany, Italy, Switzerland, Spain, Belgium, Romania, Sweden, Czechia, Denmark, Slovenia, Norway, Luxembourg, Netherlands, Ukraine, Hungary, Austria, Poland, Croatia, Portugal, Greece, Slovakia, Moldova, Republic of, Serbia, Georgia, Ireland, Estonia, Albania, Belarus, Finland, Bosnia & Herzegovina, Bulgaria, Cyprus, Latvia, Lithuania, Moldova, Malta, San Marino
North America (NAm)	United States, Canada, Mexico
Rest of World (RoW)	Argentina, Chile, Brazil, Bolivia, Suriname, Colombia, Uruguay, Peru, Venezuela, Aruba, Paraguay, Ecuador, Zambia, Namibia, Zimbabwe, Mali, Angola, Ethiopia, Cameroon, Malawi, Senegal, Eritrea, Sudan, Kenya, Libya, Niger, Tunisia, Algeria, Lesotho, Morocco, South Africa, Somalia, Djibouti, Gabon, Swaziland, Cote d'Ivoire, Mozambique, Congo - Kinshasa, Congo - Brazzaville, Democratic Republic of the Congo, Western Sahara, Nigeria, Ghana, Guinea, Madagascar, Cape Verde, Côte d'Ivoire, Egypt, Mauritania, Mauritius, Mayotte, Togo, Tanzania, Australia, New Zealand, Papua New Guinea, New Caledonia, Timor-Leste, Nicaragua, Belize, Costa Rica, El Salvador, Guatemala, Honduras, Panama, Cuba, Anguilla, Antigua & Barbuda, Bahamas, Barbados, Bermuda, Cayman Islands, Dominican Republic, Grenada, St. Helena, Guadeloupe, Haiti, Jamaica, Puerto Rico, Trinidad & Tobago, French Polynesia, Marshall Islands, Guam, Vanuatu, Tuvalu, Cook Islands, Fiji, Palau, Pitcairn Islands, Tonga, Northern Mariana Islands, Maldives, Seychelles, Antarctica, Greenland, Iceland, United States Minor Outlying Islands (the), Svalbard & Jan Mayen, French Southern Territories

Table S7: Definitions of equal-length time bins

Bin	Stages Included	LAD	FAD)	Bin Midpoint	Duration
Cen6	Tortonian, Messinian, Zanclean, Piacenzian, Gelasian, Calabrian, Chibanian, Late Pleistocene	0.0117	11.630	5.82085	11.6183
Cen5	Aquitanian, Burdigalian, Langhian, Serravallian	11.6300	23.030	17.33000	11.4000
Cen4	Rupelian, Chattian	23.0300	33.900	28.46500	10.8700
Cen3	Bartonian, Priabonian	33.9000	41.200	37.55000	7.3000
Cen2	Ypresian, Lutetian	41.2000	56.000	48.60000	14.8000
Cen1	Danian, Selandian, Thanetian	56.0000	66.000	61.00000	10.0000
K8	Maastrichtian	66.0000	72.100	69.05000	6.1000
K7	Campanian	72.1000	83.600	77.85000	11.5000
K6	Turonian, Coniacian, Santonian	83.6000	93.900	88.75000	10.3000
K5	Cenomanian	93.9000	100.500	97.20000	6.6000
K4	Albian	100.5000	113.000	106.75000	12.5000
K3	Aptian	113.0000	121.400	117.20000	8.4000
K2	Hauterivian, Barremian	121.4000	132.600	127.00000	11.2000
K1	Berriasian, Valanginian	132.6000	145.000	138.80000	12.4000
J6	Tithonian	145.0000	149.200	147.10000	4.2000
J5	Callovian, Oxfordian, Kimmeridgian	149.2000	165.300	157.25000	16.1000
J4	Bajocian, Bathonian	165.3000	170.900	168.10000	5.6000
J3	Toarcian, Aalenian	170.9000	184.200	177.55000	13.3000
J2	Pliensbachian	184.2000	192.900	188.55000	8.7000
J1	Hettangian, Sinemurian	192.9000	201.400	197.15000	8.5000
Tr4	Norian, Rhaetian	201.4000	227.000	214.20000	25.6000
Tr3	Carnian	227.0000	237.000	232.00000	10.0000
Tr2	Anisian, Ladinian	237.0000	247.200	242.10000	10.2000
Tr1	Induan, Olenekian	247.2000	251.902	249.55100	4.7020
P4	Wuchiapingian, Changhsingian	251.9020	259.510	255.70600	7.6080
P3	Roadian, Wordian, Capitanian	259.5100	273.010	266.26000	13.5000
P2	Artinskian, Kungurian	273.0100	290.100	281.55500	17.0900
P1	Asselian, Sakmarian	290.1000	298.900	294.50000	8.8000
C5	Kasimovian, Gzhelian	298.9000	307.000	302.95000	8.1000
C4	Bashkirian, Moscovian	307.0000	323.200	315.10000	16.2000
C3	Serpukhovian	323.2000	330.900	327.05000	7.7000
C2	Visean	330.9000	346.700	338.80000	15.8000
C1	Tournaisian	346.7000	358.900	352.80000	12.2000
D5	Famennian	358.9000	372.200	365.55000	13.3000
D4	Frasnian	372.2000	382.700	377.45000	10.5000
D3	Eifelian, Givetian	382.7000	393.300	388.00000	10.6000
D2	Emsian	393.3000	407.600	400.45000	14.3000
D1	Lochkovian, Pragian	407.6000	419.200	413.40000	11.6000
S2	Sheinwoodian, Homerian, Gorstian, Ludfordian, Pridoli	419.2000	433.400	426.30000	14.2000
S1	Rhuddanian, Aeronian, Telychian	433.4000	443.800	438.60000	10.4000
Or5	Katian, Hirnantian	443.8000	453.000	448.40000	9.2000
Or4	Sandbian	453.0000	458.400	455.70000	5.4000
Or3	Dapingian, Darriwilian	458.4000	470.000	464.20000	11.6000
Or2	Floian	470.0000	477.700	473.85000	7.7000
Or1	Tremadocian	477.7000	485.400	481.55000	7.7000
Cm4	Paibian, Jiangshanian, Stage 10	485.4000	497.000	491.20000	11.6000
Cm3	Wuliuan, Drumian, Guzhangian	497.0000	509.000	503.00000	12.0000
Cm2	Stage 2, Stage 3, Stage 4	509.0000	529.000	519.00000	20.0000
Cm1	Fortunian	529.0000	538.800	533.90000	9.8000
